# Gut Microbiota‐Butyrate‐PPARγ Axis Modulates Adipose Regulatory T Cell Population

**DOI:** 10.1002/advs.202411086

**Published:** 2025-02-25

**Authors:** Banru Chen, Lizhi Guan, Chao Wu, Yiwen Gong, Lei Wu, Minchun Zhang, Zhiwen Cao, Yufei Chen, Chengcan Yang, Bing Wang, Yunqi Li, Bin Li, Yufang Bi, Guang Ning, Jiqiu Wang, Weiqing Wang, Ruixin Liu

**Affiliations:** ^1^ Department of Endocrine and Metabolic Diseases Shanghai Institute of Endocrine and Metabolic Diseases Ruijin Hospital Shanghai Jiao Tong University School of Medicine Shanghai 200025 China; ^2^ Shanghai National Clinical Research Center for Metabolic Diseases Key Laboratory for Endocrine and Metabolic Diseases of the National Health Commission of the PR China Shanghai Key Laboratory for Endocrine Tumor State Key Laboratory of Medical Genomics Ruijin Hospital Shanghai Jiao Tong University School of Medicine Shanghai 200025 China; ^3^ Department of General Surgery Shanghai Ninth People's Hospital Shanghai Jiao Tong University School of Medicine 639 Zhizaoju Road Shanghai 200011 China; ^4^ Shanghai Institute of Hematology State Key Laboratory of Medical Genomics National Research Center for Translational Medicine at Shanghai Ruijin Hospital Shanghai Jiao Tong University School of Medicine 197 Ruijin 2nd Road Shanghai 200025 China; ^5^ Department of Immunology and Microbiology Shanghai Institute of Immunology Shanghai Jiao Tong University School of Medicine Shanghai 200025 China

**Keywords:** butyrate, fiber, gut microbiota, PPARγ, ST2, Treg, visceral adipose tissues (VATs)

## Abstract

Gut microbiota is essential for the function of peripherally‐induced regulatory T (pTreg) cells. However, how commensal bacteria affect thymically derived fat‐resident Treg cells that harbor a unique expression of peroxisome proliferator‐activated receptor (PPAR)‐γ and suppress inflammation in visceral adipose tissue (VAT), is not well defined. Here it is revealed that microbiota depletion causes a drastic decline in Treg cell population in VAT, particularly those expressing ST2 (ST2^+^ Treg), which are largely restored after gut microbiome reconstruction. Mechanistically, gut microbiota‐derived butyrate increases VAT ST2^+^ Treg cells through binding PPARγ. Butyrate supplementation and high fiber diet increase VAT ST2^+^ Treg population in obese mice, and ameliorated glucose tolerance and visceral inflammation. Furthermore, human omental adipose Treg cells show positive correlation with fecal butyrate and certain butyrate‐producing microbes. This study identifies the critical role of gut microbiota‐butyrate‐PPARγ axis in maintaining VAT Treg population, pinpointing a potential approach to augment VAT Treg population and ameliorate inflammation.

## Graphical abstract

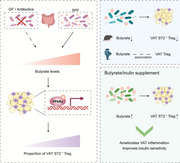

## 1 Introduction

The prevalence of obesity is increasing worldwide, being a major health challenge associated with chronic inflammation, a common denominator of systemic metabolic disorders.^[^ ^1^ ^]^ Visceral adipose tissue (VAT) harbors a wide range of immune cell populations and is one of the key sources of inflammatory mediators driving metabolic dysregulation and systematic chronic inflammation.^[^ ^2^ ^]^ Investigations on factors regulating immune cells resident in VAT are of significance for controlling inflammation and maintaining adipose homeostasis, potentially ushering approaches toward anti‐inflammatory therapeutics for metabolic syndrome.

Varies of immune cell populations in VAT are involved in metabolic regulation. Among them, Foxp3‐expressing regulatory T (Treg) cells act as a key defender of the anti‐inflammatory state in VAT. In obese individuals, changes in excess lipid storage and factors like lipotoxicity, hypoxia, and necrosis create a pro‐inflammatory microenvironment, in which Treg cells decline, and macrophages shift from an alternative (M2‐like) phenotype to a pro‐inflammatory (M1‐like) state.^[^ ^2^, ^3^ ^]^ However, the stimuli or factors that shape the VAT Treg cell population are still largely undetermined.

Gut microbiota and its metabolites have emerged as important regulators of immune system. Various immune cell types and cytokine profiles in intestinal lamina propria have been demonstrated to be regulated by gut microbiota.^[^ ^3b^ ^]^ For instance, gut microbiota plays a crucial role in the generation of peripherally induced Treg (pTreg) cells.^[^ ^4^ ^]^ These cells are negative for Helios or Neurophilin‐1, two markers that distinguish thymus‐derived Tregs (tTregs) from pTreg.^[^ ^5^ ^]^ Specifically, microbiota‐derived short‐chain fatty acids (SCFAs) especially butyrate promote colonic pTreg differentiation by enhancing histone H3 acetylation at the promoter and conserved noncoding sequence 1 (CNS1) of the *Foxp3* locus.^[^ ^6^ ^]^ Furthermore, several secondary bile acids such as isodeoxycholic acid and isoallolithocholic acid enhance the differentiation of colonic retinoic‐acid‐receptor‐related orphan nuclear receptor‐γt (RORγt^+^) pTreg through the activation of CNS1 and the production of mitochondrial reactive oxygen species, respectively.^[^ ^7^ ^]^

Unlike colonic peripheral RORγ^+^ Treg cells, VAT Treg cells are thymus‐derived and exhibit a distinct phenotype characterized by the expression of peroxisome proliferator‐activated receptor‐γ (PPAR‐γ).^[^ ^8^ ^]^ VAT Treg cells play an important role in maintaining immune homeostasis and insulin sensitivity.^[^ ^9^ ^]^ Recent advances in single‐cell RNA sequencing (scRNA‐seq) have further revealed heterogeneity within VAT Treg cells.^[^ ^10^ ^]^ These studies identify two distinct subsets based on the expression of the interleukin‐33 receptor (ST2): ST2^+^ (or ST2^high^) and ST2^−^ (ST2^low^) Treg subsets. The ST2^+^ subset is characterized by high expression of PPARγ, a transcription factor critical for its development and function. Functional studies have demonstrated that PPARγ is indispensable for the generation of ST2^+^ VAT Treg cells, as its deletion selectively abolishes ST2^+^ subset.^[^ ^10^ ^]^ The accumulation of VAT Treg cells is influenced by age, sex, and obese status.^[^ ^11^ ^]^ However, it remains unknown whether commensal bacteria could affect VAT Treg cells or their subsets.

Here, we focus on the unique features of immune cells residing in VAT in the presence or absence of gut microbiota. scRNA‐seq reveals a marked reduction in VAT Treg cells upon microbiota depletion. Microbiota deprivation and reconstitution models confirm that gut microbiota is indispensable in maintaining ST2‐expressing (ST2^+^) Tregs in VAT. We further identify that butyrate acting as an endogenous PPARγ agonist directly binds to PPARγ and promotes PPARγ‐mediated transcriptional activity, thereby increasing VAT ST2^+^ Treg cell population. Depletion of PPARγ in Treg cells largely abolishes the effects of butyrate, indicating the importance of butyrate‐PPARγ axis in the maintenance of VAT ST2^+^ Treg cells. Therapeutically, butyrate and inulin supplementation effectively increase ST2^+^ Treg cells and alleviate chronic inflammation in VAT during obesity. In humans, fecal butyrate levels correlate with Treg cells in omental adipose tissue. Together, our findings suggest that gut microbiota and its microbial fermentation product, butyrate, is essential for maintaining VAT Treg cells through activating PPARγ.

## 2 Results

### 2.1 Gut Microbiota Is Indispensable for the Maintenance of ST2^+^ Treg Cells in VAT

**Figure 1:**
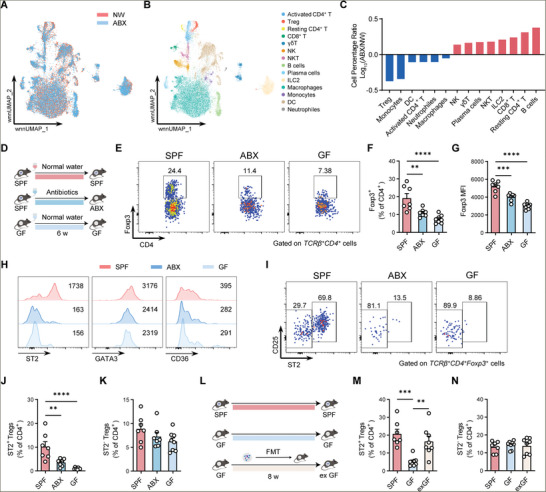
Gut microbiota is essential for the maintenance of ST2^+^ Treg cells in VAT. A,B) scRNA‐seq data of VAT CD45^+^ cells from 15‐week‐old SPF mice treated with normal water (NW) and antibiotics (ABX). A) Uniform Manifold Approximation and Projection (UMAP) representation of single cells from the two indicated groups or B) the cell types identified by marker genes. UMAP was obtained based on both scRNA‐seq and AbSeq Ab‐Oligos (antibody‐oligonucleotides). For (B), distinct clusters are annotated and color‐coded. C) Ratios of the distinct immune cell percentage of ABX mice to that of NW mice. D) Schematic of the experiment presented in (E–K). Representative of 15‐week‐old male SPF (*n* = 7), ABX (*n* = 7), and GF (*n* = 8) mice. E) Representative flow‐cytometric plots of Foxp3^+^ Treg cells among VAT TCRβ^+^CD4^+^ cells. Numbers in the plots indicate the percentages of Foxp3^+^ Treg cells among VAT TCRβ^+^ CD4^+^ cells. F) The percentages of Foxp3^+^ Treg cells among VAT CD4^+^ cells. G) The mean fluorescence intensity (MFI) of Foxp3 among VAT Foxp3^+^ Treg cells. H) Representative flow‐cytometric histograms of indicated cell markers ST2, GATA3, and CD36 among VAT Foxp3^+^ Treg cells. Numbers in the histograms indicate the MFI of these indicated markers among VAT Foxp3^+^ Treg cells. I) Representative flow‐cytometric plots of ST2^+^ CD25^+^ cells among VAT TCRβ^+^ CD4^+^ Foxp3^+^ cells. Numbers in the plots indicate the percentages of ST2^+^ Treg cells or ST2^−^ Treg cells among VAT Foxp3^+^ Treg cells. J,K) The percentages of ST2^+^ Treg cells (J) and ST2^−^ Treg cells (K) among VAT CD4^+^ cells. L–N) The experimental schematic (L), the percentages of ST2^+^ Treg cells (M), and ST2^−^ Treg cells (N) among VAT CD4^+^ cells are shown. SPF mice, *n* = 7; GF mice, *n* = 8; exGF mice, *n* = 8. Data are shown as the mean ± s.e.m. Statistical differences between groups were assessed by one‐way ANOVA with Dunnett's multiple comparisons test. *p < 0.05, **p < 0.01, ***p < 0.001, ****p < 0.0001.

The ex‐organ influence of gut microbiota on visceral adipose immune homeostasis has not been illustrated previously. We performed scRNA‐seq of CD45^+^ immune cells in VAT from conventionally raised specific pathogen‐free (SPF) and antibiotic‐treated (ABX) mice, to comprehensively understand the impact of gut microbiota on adipose immune cells. We clustered immune cells into 14 subsets based on the selected most variable genes and canonical immune cell surface markers (**Figure** **1**A,B; Figure S1, Supporting Information). Most immune cell subsets were retained or increased to varying degrees after gut microbiota depletion, while notably, the proportion of Foxp3‐expressing Treg cells was most strikingly decreased in ABX mice (Figure 1C). The decreased ratio of visceral Treg cells in ABX group suggested a potential role of gut microbiota in regulating visceral Treg cell population. We then performed flow cytometry to verify changes of VAT Treg cells in germ‐free (GF) and ABX mice, and confirmed the significant decrease in VAT Treg cells in gut microbiota‐deficient mice (Figure 1D–F). Moreover, the remaining VAT Treg cells also had decreased expression of Foxp3, suggesting a general loss of Treg cell functionality (Figure 1G). Specifically, the remaining adipose Treg cells in microbiota‐depleted mice expressed dramatically low levels of the IL‐33 receptor ST2, whereas the expression of other typical VAT Treg markers, GATA3, and CD36, was largely preserved (Figure 1H). It has been reported that Treg cells can be classified into two subsets: ST2^+^ and ST2^−^.^[^ ^10^ ^]^ We further analyzed the changes of ST2^+^ and ST2^−^ adipose Treg subsets based on ST2 expression (Figure 1I). Strikingly, we found that microbiota deletion led to a significant decrease in the proportion and total number of ST2^+^ Treg cells but not in that of ST2^−^ Treg subset in VAT (Figure 1J,K; Figure S2A–D, Supporting Information). Unlike VAT, Treg proportions in SAT did not significantly decline after microbiota depletion (Figure S2E,F, Supporting Information), while specifically, the proportion of ST2^+^ Tregs in SAT showed a reduction in GF mice but not in antibiotic‐treated mice (Figure S2G, Supporting Information). Notably, the proportion of ST2^+^ Tregs was much higher in VAT compared to SAT (Figure S2H, Supporting Information). These findings emphasize the more robust responsiveness of VAT Tregs to microbiota changes. We next examined whether Treg cells in the spleen were also affected by gut microbiota, and found that the proportion and total number of splenic Treg populations did not differ upon microbiota depletion (Figure S2I–L, Supporting Information), indicating that the changes in VAT Treg cells in response to microbiota depletion did not arise from the spleen. Furthermore, we performed fecal microbiota transplantation (FMT) in GF mice to reconstitute the gut microbiota‐enriched microenvironment. Consequently, ST2^+^ adipose Treg cell proportion was largely recovered after microbiota recolonization, whereas ST2^−^ adipose Treg cells were stable across microbiota changes (Figure 1L–N). Taken together, these data indicate a vital role of gut microbiota in maintaining visceral Treg cells.

### 2.2 Butyrate‐Producing Microbiota Is Correlated with VAT ST2^+^ Treg Cell Maintenance

**Figure 2:**
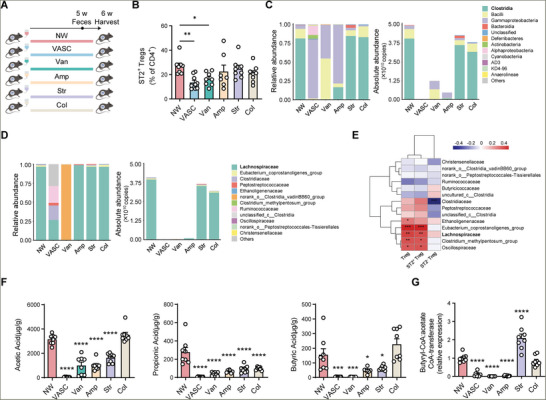
Depletion of butyrate‐producing microbiota correlates with the reduction of ST2^+^ Treg in VAT. A) Schematic of the experiment presented in (B–G). B) The percentages of ST2^+^ Treg cells among VAT CD4^+^ cells from 15‐week‐old male mice treated with NW (*n* = 8), antibiotic cocktail (VASC) (*n* = 9), vancomycin (Van) (*n* = 8), ampicillin (Amp) (*n* = 7), streptomycin (Str) (*n* = 8), and colistin (Col) (*n* = 8). C,D) Relative abundance (left) and absolute abundance (right) of bacterial taxa at the class level (C) or the family level (D) through 16S rRNA analysis. E) Heatmap of the Spearman's correlation coefficients between the percentages of Treg, ST2^+^ Tregs, and ST2^−^ Tregs among VAT CD4^+^ cells and gut bacteria at family level as assessed by 16S rRNA absolute quantification. F) Acetic acid, propionic acid, and butyric acid concentrations in fecal samples of mice treated with NW and each indicated antibiotic. NW, *n* = 8, VASC, *n* = 8, Van, *n* = 8, Amp, *n* = 7, Str, *n* = 8 and Col, *n* = 8. G) Relative gene expression of butyryl‐CoA: acetate CoA‐transferase in fecal samples of mice treated with NW and each indicated antibiotic. NW, *n* = 8, VASC, *n* = 7, Van, *n* = 8, Amp, *n* = 8, Str, *n* = 8 and Col, *n* = 8. Data are shown as the mean ± s.e.m. Statistical differences between groups were assessed by Kruskal‐Wallis test with Dunn's multiple comparisons test (B) and one‐way ANOVA with Dunnett's multiple comparisons test (F,G). *p < 0.05, **p < 0.01, ***p < 0.001, ****p < 0.0001.

Next, we seek to screen the key microbes that are essential for VAT Treg cell maintenance. Mice were given a broad‐spectrum antibiotic cocktail containing ampicillin, vancomycin, streptomycin, and colistin or each individual antibiotic in drinking water for gut microbiota depletion, where each antibiotic modulated microbiota in a distinct way, to determine the specific microbes associated with visceral ST2^+^ adipose Treg cells. As a result, vancomycin‐treated mice showed a significant reduction in ST2^+^ adipose Treg cells to a similar degree as the broad‐spectrum antibiotic cocktail group, indicating that vancomycin‐sensitive microbiota may act as an important factor in maintaining ST2^+^ adipose Treg cells (**Figure** **2**A,B).

Vancomycin treatment resulted in a nearly complete depletion in the abundance of *Clostridia* (Figure 2C; Figure S3A, Supporting Information). Further analysis of the taxa from *Clostridia* revealed that vancomycin‐treated mice showed the maximum decrease in the absolute and relative abundance of family‐level taxa known to produce SCFAs (Figure 2D; Figure S3B, Supporting Information), such as *Lachnospiraceae*, a typical butyrate‐producing microbe.^[^ ^12^ ^]^ Vancomycin treatment also resulted in a significant decrease in alpha diversity and beta diversity compared with control group (Figure S3C,D, Supporting Information). Meanwhile, the abundance of *Lachnospiraceae* showed positive correlations with the frequency of Treg and ST2^+^ Treg cells in VAT (Figure 2E). We next validated that several bacteria species that are reported to produce butyrate, including *Clostridium symbiosum*,^[^ ^12^ ^]^ *Roseburia intestinalis*,^[^ ^12^ ^]^ *Coprococcus eutactus*,^[^ ^12^ ^]^ *Anaerostipes hadrus*,^[^ ^12^ ^]^ *Coprococcus comes*,^[^ ^12^ ^]^ *Roseburia faecis*,^[^ ^13^ ^]^ *Blautia massiliensis*,^[^ ^14^ ^]^ *Blautia wexlerae*,^[^ ^14^ ^]^ *Lachnospira pectinoschiza*,^[^ ^14^ ^]^ and *Fusicatenibacter saccharivorans* ^[^ ^15^ ^]^ from *Lachnospiraceae*, and *Intestinimonas butyriciproducens* ^[^ ^16^ ^]^ and *Faecalibacterium prausnitzii* ^[^ ^17^ ^]^ in other family from *Clostridia*, were dramatically decreased due to vancomycin treatment and showed a positive correlation with ST2^+^ Treg cell frequency in the VAT (Figure S3E,F, Supporting Information).

Consistent with the reduction of butyrate‐producing bacteria, we also detected a significant drop in the fecal butyrate concentration in vancomycin group, and to a less extent, two other SCFAs, acetate and propionate were also reduced (Figure 2F). Meanwhile, bacterial genes encoding butyryl‐CoA: acetate CoA‐transferase, an enzyme involved in butyrate production by lactate‐utilizing bacteria in the gut, was also largely decreased in the feces after vancomycin treatment (Figure 2G). These data uncover the correlation of butyrate‐producing bacteria and butyrate with ST2^+^ adipose Treg cell maintenance, suggesting the possibility of butyrate as a potential mediator for regulating ST2^+^ adipose Treg cell population.

### 2.3 Butyrate Augments VAT Treg Cells via PPARγ Activation

**Figure 3:**
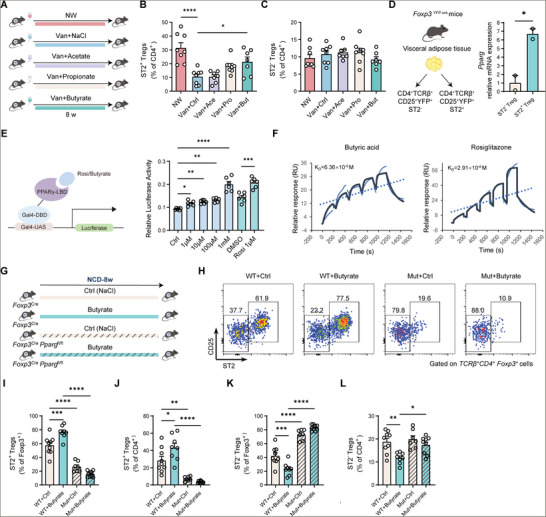
Butyrate supplementation increases VAT ST2^+^ Treg cells by promoting PPARγ activity. A) Schematic of the experiment presented in (B,C). B,C) The percentages of ST2^+^ Treg cells (B) and ST2^−^ Treg cells (C) among VAT CD4^+^ cells from 15‐week‐old male mice treated with NW (*n* = 8), Van+NaCl (*n* = 8), Van+Acetate (*n* = 7), Van+Propionate (*n* = 8), and Van+Butyrate (*n* = 7). D) The experimental schematic (left) and relative mRNA levels of *Pparg* in ST2^+^ and ST2^−^ VAT Treg cells (n  =  2 per group with each biological replicate containing pooled ST2^+^ or ST2^−^ VAT Treg cells from 14 mice). E) The experimental schematic (left) and PPARγ‐mediated luciferase reporter assay (right) in HEK293 cells, treated with a PPARγ activator rosiglitazone (1 µM), butyrate (1 µM, 10 µM, 100 µM, and 1mM) or DMSO. The relative luciferase activity was determined by the fold ratio of firefly luciferase to Renilla luciferase activity. LBD, ligand‐binding domain; DBD, DNA binding domain; UAS, upstream activating sequence. F) Sensorgrams for the affinity of butyric acid (left) and rosiglitazone (right) with PPARγ by SPR assays. G) Schematic of the experiment presented in (H‐L). *n* = 9 for *Foxp3* ^Cre^ (WT) +Ctrl and *Foxp3* ^Cre^ *Pparg* ^fl/fl^ (Mut) +Butyrate groups, *n* = 8 for *Foxp3* ^Cre^ (WT) +Butyrate group, and *n* = 7 for *Foxp3* ^Cre^ *Pparg* ^fl/fl^ (Mut) +Ctrl group. H) Representative flow‐cytometric plots of ST2^+^ CD25^+^ cells among VAT TCRβ^+^ CD4^+^ Foxp3^+^ cells. Numbers in the plots indicate the percentages of ST2^+^ Treg cells or ST2^−^ Treg cells among VAT Foxp3^+^ Treg cells. I,J) The percentages of ST2^+^ Treg cells among VAT Foxp3^+^ Treg cells (I) or among CD4^+^ cells (J) are shown. K,L) The percentages of ST2^−^ Treg cells among VAT Foxp3^+^ Treg cells (K) or among CD4^+^ cells (L) are shown. Data are shown as the mean ± s.e.m. Statistical differences between groups were assessed by one‐way ANOVA with Dunnett's multiple comparisons test (B,C,E), two‐tailed unpaired Student's t‐tests (D) and two‐way ANOVA with Šidák's multiple comparison test (I–L). *p < 0.05, **p < 0.01, ***p < 0.001, ****p < 0.0001.

SCFAs have been reported to be crucial in regulating colonic Treg cell homeostasis.^[^ ^6^, ^18^ ^]^ To assess the effects of butyrate and two other SCFAs on VAT Treg cells, we treated SPF mice with vancomycin in combination with butyrate, acetate, or propionate in the drinking water (**Figure** **3**A). Consistently, vancomycin treatment resulted in a drastic decrease in the proportion of ST2^+^ adipose Treg cells, while the provision of butyrate to vancomycin‐treated mice augmented ST2^+^ adipose Treg cells. Acetate and propionate did not show similar effects (Figure 3B). Meanwhile, the proportion of ST2^−^ Treg cells remained stable across different treatments (Figure 3C).

VAT Treg cells are primarily derived from the thymus (tTreg) and seeded in VAT early in life, rather than being peripherally converted Treg cells.^[^ ^8^, ^11^, ^19^ ^]^ The transcription factor PPARγ has been identified as a major driver of VAT Treg cells.^[^ ^8a^ ^]^ A recent study further reports that PPARγ is specifically highly expressed in ST2^+^ adipose Treg cell subset compared to ST2^−^ Treg subset, as revealed by scRNA‐seq.^[^ ^10^ ^]^ We next sorted VAT ST2^+^ and ST2^−^ Treg cells and confirmed that *Pparg* mRNA levels were much higher in ST2^+^ Treg cells compared to ST2^−^ Treg cells (Figure 3D). These findings suggest that PPARγ may act as a possible mediator linking butyrate and ST2^+^ adipose Treg cells. To assess the ability of butyrate to activate PPARγ‐mediated transcription, HEK293 cells were transfected with a fusion construct containing the PPARγ‐ligand binding domain (LBD) and Gal4‐DNA binding domain (DBD) and incubated with an increasing concentration of butyrate (Figure 3E). Notably, PPARγ‐mediated transcription was significantly activated by butyrate in a dose‐dependent manner (Figure 3E). Surface plasmon resonance (SPR) measurements further revealed the direct binding of butyric acid or butyrate to PPARγ protein with an estimated dissociation constant (*k_D_*) value of 63.6 µm and 119µm, respectively, while acetate and propionate showed no capacity to bind to PPARγ protein (Figure 3F; Figure S4, Supporting Information). These data support that butyrate can directly interact with PPARγ and activate PPARγ‐mediated transcriptional activity.

To investigate the role of PPARγ in the regulatory effects of butyrate on ST2^+^ adipose Treg cells, we specifically deleted PPARγ in Treg cells by cross‐breeding the *Foxp3* ^Cre^ strain with *Pparg* ^fl/fl^ mice. Treg‐*Pparg* knockout mice and their littermate controls were treated with butyrate‐containing drinking water or pH and sodium‐matched water as controls (Figure 3G). ST2^+^ adipose Treg cells showed a sharp decline in Treg‐*Pparg* knockout mice (*Foxp3* ^Cre^ *Pparg* ^fl/fl^) than control mice, whereas the proportion of ST2^−^ adipose Treg cells was not affected by PPARγ deficiency (Figure 3H–L), consistent to the driver effect of PPARγ on adipose Treg cells.^[^ ^8a^ ^]^ Importantly, we observed a significant increase in the proportion of ST2^+^ adipose Treg cells after butyrate treatment in control mice, while butyrate failed to induce the enrichment of ST2^+^ adipose Treg cells in Treg‐*Pparg* knockout mice (Figure 3I,J). Together, our data demonstrate that PPARγ acts as a binding protein of butyrate and mediates its enhancing effects on ST2^+^ adipose Treg cells.

### 2.4 Butyrate Increases VAT ST2^+^ Treg Cells and Ameliorates Metabolic Inflammation in Obese Mice via PPARγ

**Figure 4:**
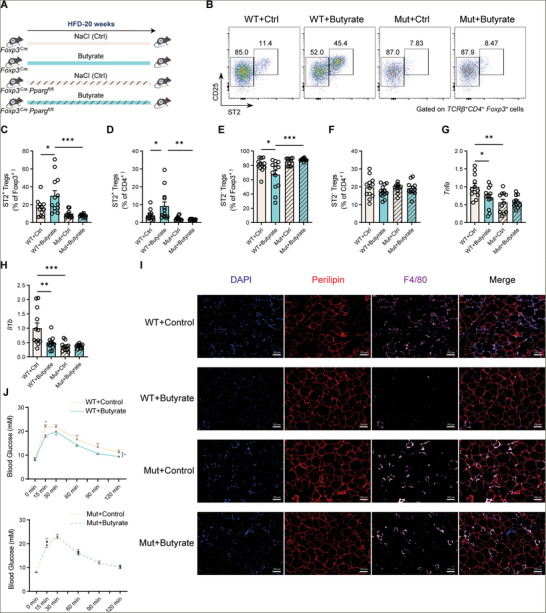
Butyrate supplementation increases VAT ST2^+^ Treg cells and ameliorates adipose inflammation in obese mice. A) Schematic of the experiment presented in (B‐J). *n* = 12–13 for *Foxp3* ^Cre^ (WT) +Ctrl group; *n* = 12–13 for *Foxp3* ^Cre^ (WT) +Butyrate group; *n* = 11 for *Foxp3* ^Cre^ *Pparg* ^fl/fl^ (Mut) +Ctrl and *n* =11–12 for *Foxp3* ^Cre^ *Pparg* ^fl/fl^ (Mut) +Butyrate group. B) Representative flow‐cytometric plots of ST2^+^ CD25^+^ cells among VAT TCRβ^+^ CD4^+^ Foxp3^+^ cells. Numbers in the plots indicate the percentages of ST2^+^ Treg cells or ST2^−^ Treg cells among VAT Foxp3^+^ Treg cells. C,D) The percentages of ST2^+^ Treg cells among VAT Foxp3^+^ cells (C) and CD4^+^ cells (D) are shown. E,F) The percentages of ST2^−^ Treg cells among VAT Foxp3^+^ cells (E) and CD4^+^ cells (F) are shown. G,H) Relative RNA expression of *Tnfa* (G) and *Il1b* (H) in VAT. I) Representative immunofluorescence staining of perilipin1 and F4/80 in VAT from four groups. Images are representative of four independent samples, scale bar 100 µm. J) Top, glucose tolerance tests (GTT) for WT+Control and WT+Butyrate group. Bottom, GTT for Mut+Control and Mut+Butyrate group. Data are shown as the mean ± s.e.m. Statistical differences between groups were assessed by two‐way ANOVA with Šidák's multiple‐comparisons test (C–H) and two‐way repeated‐measures ANOVA with Bonferroni's multiple comparisons test (J). *p < 0.05, **p < 0.01, ***p < 0.001, ****p < 0.0001.

We next examined whether butyrate could restore VAT ST2^+^ Treg cells in pathological conditions in which VAT ST2^+^ Treg cells decline. It has been reported that VAT Treg cell population shows a substantial reduction in diet‐induced obesity,^[^ ^9^, ^20^ ^]^ and VAT Treg cells are known to improve obesity‐associated inflammation and insulin resistance.^[^ ^9^ ^]^ Therefore, we further investigated whether butyrate could affect VAT Treg cells and metabolic inflammation in high‐fat diet (HFD)‐induced obese mice and whether this effect was via the butyrate‐PPARγ axis. We treated *Foxp3* ^Cre^ *Pparg* ^fl/fl^ mice and their littermate controls with butyrate or NaCl in drinking water throughout the development of diet‐induced obesity (**Figure** **4**A). Butyrate treatment significantly increased the percentage of ST2^+^ Treg cells in VAT of control mice but not in *Foxp3* ^Cre^ *Pparg* ^fl/fl^ mice (Figure 4B–F). Importantly, butyrate‐treated control mice showed amelioration of HFD‐induced adipose inflammation, including reduced expression of tissue inflammation markers, such as *Il1b* and *Tnfa*, and less pro‐inflammatory cell infiltration, whereas these effects of butyrate were largely ablated in *Foxp3* ^Cre^ *Pparg* ^fl/fl^ mice (Figure 4G–I). Furthermore, butyrate treatment also improved glucose tolerance in control mice fed HFD but not in *Foxp3* ^Cre^ *Pparg* ^fl/fl^ mice (Figure 4J). These results indicate the importance of a butyrate‐PPARγ axis in VAT Treg cells during obesity. Together, butyrate treatment can retain ST2^+^ Treg cells in VAT during HFD and ameliorate HFD‐induced inflammation via PPARγ.

### 2.5 Fiber Supplementation Increases ST2^+^ Treg Cells and Improves Chronic Inflammation in VAT during Diet‐Induced Obesity

**Figure 5:**
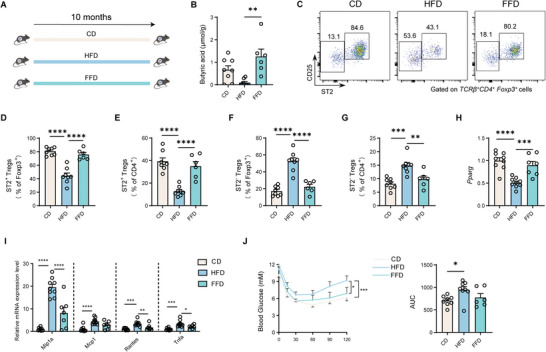
Dietary fiber increases Butyrate and VAT Treg levels and ameliorates obesity‐related inflammation. A) Schematic of the experiment. B) Butyric acid concentrations in fecal samples of mice treated with each diet. C) Representative flow‐cytometric plots of ST2^+^ CD25^+^ cells among VAT TCRβ^+^ CD4^+^ Foxp3^+^ cells. Numbers in the plots indicate the percentages of ST2^+^ Treg cells or ST2^−^ Treg cells among VAT Foxp3^+^ Treg cells. D, E) The percentages of ST2^+^ Treg cells among VAT Foxp3^+^ cells (D) and CD4^+^ cells (E) are shown. F, G) The percentages of ST2^−^ Treg cells among VAT Foxp3^+^ cells (F) and CD4^+^ cells (G) are shown. H) Relative mRNA expression of *Pparg* in VAT of different groups. I) Relative RNA expression of *Mip1a*, *Mcp1*, *Rantes*, and *Tnfa* in VAT of different groups. J) Insulin tolerance tests (ITT) for three groups. For B–G, J), *n* = 8 for control group and HFD group; *n* = 6 for FFD group. For H‐I, *n* = 9 for control group and HFD group; *n* = 7 for FFD group. Data are shown as the mean ± s.e.m. Statistical differences between groups were assessed by one‐way ANOVA with Šídák's multiple comparisons test (B, D–I, J(right)) and two‐way repeated‐measures ANOVA with Dunnett's multiple comparisons test (J, left). *p < 0.05, **p < 0.01, ***p < 0.001, ****p < 0.0001.

Inulin, a slowly digestible fructose polymer, can be effectively converted into SCFAs by gut microbiota.^[^ ^21^ ^]^ We next investigated whether supplementation with dietary fiber containing inulin, a more practical and lifestyle‐friendly approach that could be fermented to produce butyrate, can also enhance ST2^+^ Treg cells and ameliorate chronic inflammation in adipose tissues in diet‐induced obesity. Mice were fed with chow diet (CD), HFD and HFD supplemented with inulin as a source of fiber (high‐fat/high‐fiber diet, FFD) (**Figure** **5**A).^[^ ^22^ ^]^ Following the inulin‐enriched HFD, the concentrations of three SCFAs significantly increased in the feces, with butyrate showing the largest fold increase (Figure 5B; Figure S5, Supporting Information). Consistent with previous studies,^[^ ^8^, ^9^, ^10^ ^]^ we observed that ST2^+^ Treg cells were significantly decreased in adipose tissue of mice fed HFD compared with CD‐fed mice (Figure 5C–G). Importantly, inulin supplementation significantly restored the percentage of ST2^+^ Treg cells in VAT (Figure 5C–G). The expression of *Pparg* exhibited a similar changing pattern to the frequency of ST2^+^ Treg cells in VAT (Figure 5H). In addition, inulin supplementation led to a decreased expression of inflammatory genes and improved insulin sensitivity (Figure 5I,J). These results suggest that dietary fiber intake elevates butyrate concentration and increases the pool of ST2^+^ Treg cells in VAT, associated with the improvement in obesity‐associated inflammation and metabolic abnormalities after treatment.

### 2.6 Associations of Fecal Butyrate Concentration with Treg Frequency in Human Omental Fat

**Figure 6:**
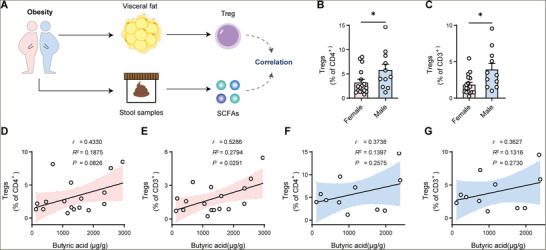
Human fecal butyrate shows a positive association with omental adipose Treg cells. A) Schematic of the experiment presented in (B–G). *n* = 17 for female obese subjects; *n* = 11 for male obese subjects. B,C) The percentages of Treg cells to CD4^+^ cells (B) or to CD3^+^ cells (C) in female and male omental fat are shown. D,E) Correlation between fecal concentration of butyric acid and the percentages of Treg cells among CD4^+^ cells (D) or among CD3^+^ cells (E) in female omental fat. F,G) Correlation between fecal concentration of butyric acid and the percentages of Treg cells among CD4^+^ cells (F) or among CD3^+^ cells (G) in male omental fat. Error bars represent the mean ± s.e.m. (B,C). The colored area indicates 95% confidence intervals (D–G). Data are shown as the mean ± s.e.m. Statistical differences between groups were assessed by two‐tailed unpaired Student's *t*‐tests (B), Mann‐Whitney test (C), simple linear regression and Pearson's correlation (D–G).

To extend our findings into human physiology, we assessed the relationship between butyrate and VAT Treg cells in humans. Omental fat and stool samples from 11 male and 17 female obese individuals undergoing bariatric surgery for weight loss were collected (**Figure** **6**A). Treg cells isolated from fresh omental fat tissue were analyzed by flow cytometry. Clear Foxp3^+^ Treg cells were detected in both male and female individuals with varied Treg cell frequencies, but few of the human VAT Treg cells showed ST2 expression (Figure S6A,B, Supporting Information). According to a previous report of sex‐specific differences in Treg cells from VAT,^[^ ^23^ ^]^ we analyzed VAT Treg cell frequency in male and female individuals separately, where the frequency of VAT Treg is significantly higher in males than in females (Figure 6B,C). Furthermore, consistent with our findings in mice, fecal butyrate concentration exhibited positive correlations with VAT Treg cell frequency in both female and male individuals with more obvious in females than males (Figure 6D–G). We did not observe associations between fecal acetate or propionate concentration with VAT Treg frequency in the two sexes (Figure S6C–J, Supporting Information).

Furthermore, we performed metagenomic sequencing of human fecal samples, and found that several species that can produce butyrate were positively correlated with VAT Treg frequency in both females and males (Figure S7, Supporting Information). For instance, butyrate‐producing species *Roseburia faecis* was positively associated with VAT Treg frequency in females, while *Faecalibacterium prausnitzii* showed similar associations in males (Figure S7, Supporting Information). These findings further support the existence of gut microbiota‐butyrate‐Treg associations in humans. Together, these findings extend the relationship between gut microbiota, butyrate, and VAT Treg cells from mice to humans, pointing to butyrate supplement as a possible approach to improve VAT Treg cells in obese human individuals.

## 3 Discussion

Treg cells in VAT are important in the regulation of adipose tissue inflammation and insulin sensitivity.^[^ ^9^ ^]^ Specifically, ST2^+^ Treg cells, a Treg subset with high PPARγ expression in VAT, exhibit a more activated phenotype than ST2^−^ Treg cells.^[^ ^8^, ^24^ ^]^ Loss of ST2 expression in VAT Treg cells leads to impaired glucose tolerance.^[^ ^25^ ^]^ Here, we demonstrate that gut microbiota is essential for the maintenance of ST2^+^ Treg cells in VAT, which is associated with the abundance of butyrate‐producing bacteria. Mechanistically, gut microbiota‐derived butyrate acts as a novel mediator for the maintenance of VAT ST2^+^ Treg cells by promoting PPARγ activity.

Precursors of VAT Treg cells are derived from the thymus and turn on low levels of PPARγ expression in the spleen,^[^ ^8^, ^11^ ^]^ and subsequently, local mediators in VAT endow the canonical VAT Treg cell phenotype. Local antigens and environmental cues such as specific T cell receptor (TCR), IL‐33, insulin, and interferon‐α have been shown to regulate VAT Treg cells.^[^ ^8^, ^10^, ^19^, ^20^, ^24^, ^25^ ^]^ VAT Treg cells can be further classified into ST2^+^ and ST2^−^ Treg cell subsets with the ST2^+^ adipose Treg cell subset expressing a high level of PPARγ and the ST2^−^ Treg subset expressing low PPARγ.^[^ ^10^ ^]^ In this study, we, for the first time identified that gut microbiota acted as an indispensable regulator specifically for the VAT ST2^+^ Treg cell subset but not for the ST2^−^ Treg subset. Moreover, the changes in VAT ST2^+^ Treg cells were associated with butyrate‐producing commensal bacteria, and importantly, microbiota‐derived butyrate augmented VAT ST2^+^ Treg cells even in the absence of gut microbiota. These findings identify a new stimulus from the gut microbiota that drives VAT Treg cell population. However, the specific butyrate‐producing species that modulate VAT ST2^+^ Treg cells are not identified in the current study, warranting determination in future research.

PPARγ is reported to be a major driver of the accumulation of adipose tissue Treg cells and is highly expressed in the ST2^+^ Treg subset.^[^ ^8^, ^10^ ^]^ In this study, we identified butyrate as an endogenous PPARγ agonist. Previous in vitro studies report that butyrate can stimulate the transcriptional activity of PPARγ.^[^ ^26^ ^]^ Using PPARγ‐mediated luciferase reporter assay and SPR measurements, we demonstrated that butyrate, like PPARγ pharmacological agonist rosiglitazone, can directly bind to PPARγ and promote PPARγ‐mediated transcriptional activity, thereby increasing VAT ST2^+^ Treg cell population. Depletion of PPARγ in Treg cells largely abolished the effects of butyrate, indicating the importance of butyrate‐PPARγ axis in the maintenance of VAT ST2^+^ Treg cells. Previous studies also report the regulatory role of butyrate in intestinal Treg cells, in which butyrate functions as an inhibitor of histone deacetylase to regulate the Treg cell population.^[^ ^6^, ^18^ ^]^ The specific high expression of PPARγ in VAT Treg cells provided a novel mechanism underlying the regulatory role of gut microbiota‐derived butyrate on Treg cells.

In addition to its role in VAT Treg cells, PPARγ also plays pivotal roles in liver and intestinal homeostasis, which may suggest a potentially broader impact of the gut microbiota‐butyrate‐PPARγ axis on metabolic health. In the liver, PPARγ activation protects against hepatic fibrosis by preventing the transition of stellate cells into profibrogenic myofibroblasts.^[^ ^27^ ^]^ PPARγ agonists like rosiglitazone have been demonstrated to be effective in alleviating liver steatosis and improving insulin sensitivity in non‐alcoholic steatohepatitis.^[^ ^28^ ^]^ Additionally, PPARγ expression in intestinal epithelial cells (IECs) is crucial for maintaining epithelial barrier integrity^[^ ^29^ ^]^ and intestinal lipid metabolism.^[^ ^30^ ^]^ Moreover, PPARγ expression and activity in IECs reduces after antibiotic treatment.^[^ ^29^, ^30^ ^]^ A recent study indicates that butyrate restores Clostridia abundance and epithelial hypoxia in the colon by stimulating epithelial PPARγ signaling, protecting mice against sorbitol‐induced diarrhea.^[^ ^31^ ^]^ Collectively, gut microbiota‐derived butyrate may functionally affect metabolic homeostasis by targeting tissues highly expressing PPARγ.

ST2^+^ Treg cells are reported to ameliorate metabolic dysfunction in obesity^[^ ^10^, ^24^ ^]^ and VAT Treg cells have been observed to decline in obesity.^[^ ^9^ ^]^ We then examined whether butyrate could restore the decrease of VAT ST2^+^ Treg cells in obese mice. As expected, either butyrate supplementation or dietary fiber that can be fermented to produce butyrate can effectively increase VAT ST2^+^ Treg cell population in HFD‐induced obese mice and meanwhile, ameliorate adipose inflammation and glucose tolerance. Butyrate has been well proven to be beneficial in host metabolism. Oral butyrate treatment has a potential therapeutic effect on pediatric obesity and improves metabolic health.^[^ ^32^ ^]^ Of clinical significance, we detected the positive association of fecal butyrate concentration with Treg cell proportion in the omental fat of obese subjects, supporting the potential regulatory effects of butyrate on adipose Treg cells in humans.

This study has several limitations. Our data showed that ST2 expression was low or even undetectable in human VAT Treg cells, consistent with previous findings, in which ST2 expression is not detected in obese or lean omental adipose Treg cells,^[^ ^33^ ^]^ suggesting that human Treg cells may exhibit tissue‐specific characteristics distinct from those in mice. Despite the low ST2 level, PPARγ is highly expressed in human adipose tissue Treg cells like those in mice.^[^ ^33^, ^34^ ^]^ These findings suggest that butyrate may also have the potential to modulate human VAT Treg cells via PPARγ. Given the difference in VAT Treg cells between humans and mice, future experimental validation is still required to confirm this speculation. Additionally, in this study, we only used male mice to elucidate the modulation of gut microbiota and butyrate on ST2^+^ VAT Treg cells, considering that Treg cells are more abundant in male VAT than in female VAT.^[^ ^23^, ^35^ ^]^

In summary, our results identify gut microbiota and its derived butyrate as a new stimulus in maintaining ST2^+^ Treg cells in VAT by promoting PPARγ activity, extending our insights into the regulation of VAT immune homeostasis. Since VAT Treg cells play vital roles in improving adipose chronic inflammation and metabolic homeostasis, these findings may point to new modalities for augmenting VAT ST2^+^ Treg cells by targeting gut microbiota, thereby improving metabolic homeostasis.

## 4 Conclusion

This study identifies the indispensable role of gut microbiota in the maintenance of ST2^+^ adipose Treg cell population, and defines gut microbiota‐derived butyrate as an endogenous PPARγ agonist, modulating adipose Treg cell populations via promoting PPARγ activation. Butyrate or fiber supplementation restores the VAT ST2^+^ Treg population in obese mice, and ameliorates glucose tolerance and visceral inflammation.

## 5 Experimental Section

### Mice

All mouse lines were maintained on a C57BL/6J background. Male C57BL/6 was purchased from Linchang Company (Shanghai, China). *Foxp3* ^Cre^ mice (stock number, 016959) and *Pparg* ^fl/fl^ mice (stock number, 004584) were from the Jackson Laboratory. Mice were bred in groups of two to five mice per cage, with free access to food and water in SPF facilities. GF mice were bred and maintained in sterile isolators. All of the mice were housed in facilities with a 12‐h light‐dark cycle, an ambient temperature of 22–24 °C, and a humidity of 50–70%. Mice were fed a CD or an HFD containing 60 kcal% fat (Research Diet, D12492i). For dietary fiber treatment, mice were randomly allocated to one of the three following diets: control diet with 10 kcal % fat (CD, ReadyDietech, D12450J), HFD with 60 kcal % fat (ReadyDietech, D12492), and High‐fat/high‐fiber fat diet with 60 kcal % fat and 150g inulin per 886.85g as a source of fiber (FFD, ReadyDietech, RD21030802). The composition of these three diets is listed in Table S1 (Supporting Information). Irradiated sterile diets were used for all GF and FMT mice. For the antibiotic treatment experiments, mice were supplied with drinking water containing either ampicillin (1 g L⁻^1^), streptomycin (5 g L⁻^1^), colistin (1 g L⁻^1^), vancomycin (0.25 g L⁻^1^), or a mixture (1 g L⁻^1^ ampicillin, 5 g L⁻^1^ streptomycin, 1 g L⁻^1^ colistin, 0.25 g L⁻^1^ vancomycin). For the SCFAs feeding experiments, mice were treated with either sodium acetate, sodium propionate or sodium butyrate in the drinking water containing 150 mm concentration of each SCFA. The water solutions were pH‐adjusted and changed weekly. Control mice received pH and sodium‐matched water. Sample sizes of mouse experiments in our study are similar to those generally employed in the field. Exclusion criteria are pre‐established before the experiments. If done usually due to animals in abnormal condition. All of the mouse experiments were approved by the Animal Care Committee of Shanghai Jiaotong University School of Medicine (B‐2018‐011).

### Fecal Microbiota Transplantation (FMT)

A total of 1 g fecal samples were collected from 17 donor C57BL/6 mice. Feces were homogenized in 10 mL of sterile PBS using a vortex for 3 min followed by standing for 2 min. Afterward, the fecal suspensions were filtered through a 70‐µm strainer and then administered to recipient GF mice by oral gavage. The recipient GF mice were administered twice at a dose of 200 µL per mouse.

### Isolation of Lymphocytes

Adipose tissues excised from mice or humans were cut into small pieces and digested in 1 mg mL^−1^ collagenase type II (Sigma) in PBS (Meilunbio) supplemented with 1.5% bovine serum albumin (Sigma) for 30 min at 37 °C in a shaker. Cell suspensions were filtered through a sieve. The mouse spleen was filtered through a sieve after grinding. Afterward, the cell suspensions of adipose tissues and spleen were centrifuged and then resuspended with red blood cell lysis solution (Solarbio), and the stromal vascular fraction (SVF) was collected after centrifugation.

### Flow Cytometry

For mouse T lymphocytes analysis, cells were stained with anti‐mouse CD3‐BV605 (BD, 563 004, Clone: 145‐2C11), CD4‐BV711 (BD, 563 050, Clone: GK1.5), TCR‐β‐BV510 (BD, 563 221, Clone: H57‐597), CD45.2‐BUV737 (BD, 564 880, Clone: 104), CD25‐BV786 (BD, 564 368, Clone: 3C7), CD8‐PE (BD, 553 033, Clone: 53–6.7), CD36‐BB515 (BD, 565 094, Clone: CRF D‐2712), ST2‐APC (Biolegend, 146 606, Clone: DIH4), and FVS780 (BD, 565 388). For intranuclear staining, cells were fixed, permeabilized and intracellularly stained with anti‐mouse GATA3‐BB700 (BD, 566 642, Clone: L50‐823), and Foxp3‐PE‐Cy5 (eBioscience, 15‐5773‐82, Clone: FJK‐16s) according to the manufacturer's instructions (Foxp3/Transcription Factor Staining Buffer Set, Invitrogen™ eBioscience™). Finally, cells were acquired using an LSR Fortessa X‐20 flow cytometer (BD Biosciences), and data were analyzed using FlowJo software.

For human T lymphocyte analysis, cells were blocked with human Fc antibody (BD, 564 220, Clone: Fc1). Cells were then stained with anti‐human CD45‐BUV496 (BD, 750 179, Clone: HI30), CD19‐BUV615 (BD, 751 273, Clone: HIB19), CD3‐BUV805 (BD, 741 999, Clone: HIT3α), CD4‐BUV563(BD, 750 979, Clone: OKT4), CD8‐BUV661(BD, 741 627, Clone: HIT8α), ST2‐PerCP‐eFluor^TM^710 (eBioscience, 46‐9338‐42, Clone: hIL33Rcap), and FVS780 (BD, 565 388). For intranuclear staining, cells were fixed, permeabilized, and stained intracellularly with anti‐human Foxp3‐AF647 (BD, 560 045, Clone: 259D/C7) according to the manufacturer's instructions (Foxp3/Transcription Factor Staining Buffer Set, Invitrogen™ eBioscience™). Finally, cells were acquired using a Symphony A5 flow cytometer (BD Biosciences), and data were analyzed using FlowJo software.

### Butyryl‐CoA: Acetate CoA‐Transferase Quantification

Fecal DNA was extracted from fecal pellets using the QIAamp PowerFecal Pro DNA Kit (QIAGEN). Quantitative PCR was conducted using ChamQ Universal SYBR qPCR Master Mix (Vazyme). The primers are listed in Table S2 (Supporting Information). SYBR green quantitative PCR for bacterial 16S rRNA genes was performed using primers for bacteria (UniF340/UniR514) and the following cycling conditions: 95 °C for 30 s, 45 cycles of 95 °C for 5 s and 60 °C for 34 s.

### Real Time‐PCR

Total RNA was isolated from tissues using Eastep® Super Total RNA Extraction Kit (Promega), and cDNA was synthesized using HiScript III RT SuperMix for qPCR (Vazyme), according to the manufacturer's protocol. qPCR reactions were set up using the ChamQ Universal SYBR qPCR Master Mix (Vazyme) and the following cycling conditions: 95 °C for 30 s, 45 cycles of 95 °C for 5 s, and 60 °C for 34 s. Samples were run in duplicate in a single 384‐well reaction plate. The primers are listed in Table S2 (Supporting Information). Data were normalized to the Gapdh gene and analyzed using the ΔΔCT method.

### Luciferase Reporter Assay

A dual reporter plasmid (pGL4.31‐pRL‐CMV) encoding both firefly luciferase reporter plasmid (Promega pGL4.31 (luc2P/Gal4UAS/Hygro) and *Renilla* luciferase plasmid (Promega pRL‐CMV) was generated. The transcriptional activity of the fusion protein containing the PPARγ ligand‐binding domain and Gal4‐DNA‐binding domain was assessed by luciferase expression and reporter assays were conducted as previously described.^[^ ^36^ ^]^ In brief, 30000 HEK293 cells (Cell Bank of the Chinese Academy of Sciences, Shanghai, China, SCSP‐5209) per well were plated in 96‐well plates in antibiotic‐free Dulbecco's Modified Eagle Medium (Meilunbio) containing 10% fetal bovine serum and 1% GlutaMAX™ (Thermo Fisher Scientific). Then, cells were transfected with a DNA mixture containing 50 ng of plasmid encoding the firefly luciferase reporter and *Renilla* luciferase, and 50 ng of plasmid encoding the fusion protein containing the Gal4 DNA‐binding domain and the human PPARγ ligand‐binding domain in each well. Transfections were performed using Lipofectamine™ 2000 Transfection Reagent (Invitrogen) according to the manufacturer's instructions. After 8 h, butyrate (medium diluted and pH‐adjusted), DMSO, or rosiglitazone (Sigma) were added and the luciferase activity was measured 18 h later using the dual‐luciferase reporter kit (Meilunbio).

### Surface Plasmon Resonance (SPR) Analysis of PPARγ Protein and SCFAs

The binding mode of these five compounds (acetate, propionate, butyrate, butyric acid, and rosiglitazone) with PPARγ was measured using the SPR method on a Biacore 8K (GE Healthcare). Recombinant mouse PPARγ protein (Abcam, ab236330) was immobilized on a CM7 chip to maximize the resonance unit (RU) response because of the large molecule weight ratio of ligand and analytes (more than 100). These five compounds were diluted in buffer (1 x PBS + P, pH 7.4, 0.05% surfactant P20, DMSO concentration 1%) at the indicated concentration. Preliminary tests indicated sodium butyrate dissociated from protein slowly, therefore the low molecular weight single‐cycle kinetics method was preferred in the assay. Analytes were injected at 30 µL/min. Contact time and dissociation time were set as 180 s and 300 s, respectively. The response was determined as a function of time. K_D_ values were calculated with Biacore 8K Evaluation software, Version 3.0.12.

### Metabolic Studies

8‐10‐week‐old male *Foxp3* ^Cre^ and *Foxp3* ^Cre^ *Pparg* ^fl/fl^ mice were fed with HFD for 20 weeks, and treated with NaCl or butyrate in drinking water. For glucose tolerance test (GTT), 1.5 g per kilogram of body weight (g/kg) glucose was administered by intraperitoneal injection to mice after fasting for 16 h. Blood samples were obtained from the tail vein at the indicated time points, and blood glucose levels were measured before and 15, 30, 60, 90, and 120 min after glucose injection. 8‐10‐week‐old male mice were fed with CD, HFD, and FFD for ten months, respectively. For insulin tolerance test (ITT), insulin (1.25 U kg⁻^1^) was administered by intraperitoneal injection to mice after fasting for 6 h. Blood samples were obtained from the tail vein at the indicated times, and blood glucose levels were measured before and 15, 30, 60, 90, and 120 min after insulin injection.

### Immunofluorescence Staining

Tissue sections of VAT were deparaffinized with xylene, and antigen retrieval was performed with Tris‐EDTA (pH = 9.0). After washing with PBS for three times, sections were then blocked with 100µL 5% bovine serum albumin (BSA) for 20 min at room temperature. First, sections were incubated with anti‐Perilipin‐1 (CST, 9349) overnight at 4 °C. The next day, sections were detected with goat anti‐rabbit IgG H&L (HRP) (Abcam, ab6712) and Cy3 Tyramide (runnerbio, Bry‐880530). Antigen retrieval was performed with sodium citrate antigen retrieval buffer for 10 min in a microwave. After washing with PBS three times, sections were then blocked with 100 µL 5% BSA for 20 min at room temperature. And sections were then incubated with anti‐CD11b antibodies (Abcam, a13357) overnight at 4 °C, followed by detection with goat anti‐rabbit IgG H&L (HRP) (Abcam, ab6712) and CF488 Tyramide (runnerbio, Bry‐880488). Antigen retrieval was performed with sodium citrate antigen retrieval buffer for 10 min in a microwave. After washing with PBS three times, sections were then blocked with 100 µL 5% BSA for 20 min at room temperature. And sections were incubated with anti‐F4/80 (CST, 70 076) overnight at 4 °C, followed by detection with goat anti‐rabbit IgG H&L (HRP) (Abcam, ab6712) and AF 647 Tyramide (runnerbio, Bry‐880647). After staining, slides were counterstained with DAPI. Fluorescence panoramic imaging was performed using a 3DHISTECH pathological section scanner.

### Single‐Cell RNA Sequencing and Analysis

For single‐cell solution preparation, a single‐cell suspension was prepared from VAT of 15‐week‐old mice with NW or antibiotic treatment as mentioned above, and then the CD45^+^ cells were sorted using Magnetic‐activated cell sorting (MACS) separation (CD45 MicroBeads, Miltenyi Biotec) according to the manufacturer's protocol. Single‐cell solution of CD45^+^ cells was kept on ice before loading to BD Rhapsody cartridge for single‐cell transcriptome capturing.

For single‐cell transcriptome capturing, library construction, and sequencing, cells were first stained with two fluorescent dyes, Calcein AM and Draq7, for precise determination of cell concentration and viability via BD Rhapsody™ Scanner (BD Biosciences) before AbSeq antibodies labeling. Cells of each sample were stained with 8 AbSeq antibodies against major mouse immune markers (including BD™ AbSeq Oligo Rat Anti‐Mouse CD4 (940 108), BD™ AbSeq Oligo Rat Anti‐Mouse CD44 (940 114), BD™ AbSeq Oligo Rat Anti‐Mouse CD25 (940 116), BD™ AbSeq Oligo Rat Anti‐Mouse CD62L (940 122), BD™ AbSeq Oligo Hamster Anti‐Mouse TCRβ Chain (940 125), BD™ AbSeq Oligo Hamster Anti‐Mouse CD69 (940 126), BD™ AbSeq Oligo Rat Anti‐Mouse CD1d (940 171), and BD™ AbSeq Oligo Rat Anti‐Mouse CD8b.2 (940 181)) for 30 min on ice. Briefly, cells were labeled by different AbSeq antibodies respectively and washed three times with Stain Buffer (BD Biosciences, Cat. No. 554656). Labeled samples were loaded in one BD Rhapsody micro‐well cartridge and proceeded to single‐cell capture. Cell capture beads were then loaded excessively to ensure that nearly every micro‐well contained one bead, and the excess beads were washed away from the cartridge. After lysing cells with lysis buffer, cell capture beads were retrieved and washed prior to performing reverse transcription. Microbeads‐captured single‐cell transcriptome and Ab‐tagged index sequences targeting eight immune cell‐associated surface antigens were generated into cDNA library and Abseq library separately containing cell labels and unique molecular identifiers (UMI) information. All procedures were performed with BD Rhapsody cDNA Kit (BD Biosciences, Cat. No. 633773) and BD Rhapsody Whole Transcriptome Analysis (WTA) Amplification Kit (BD Biosciences, Cat No. 633802) strictly following the manufacturer's protocol. All the libraries were sequenced in a PE150 mode (Pair‐End for 150bp read) on the NovaSeq 6000 platform (Illumina).

For data processing, raw sequencing reads of the cDNA library were processed through the BD Rhapsody Whole Transcriptome Assay Analysis Pipeline (v1.8), which included filtering by reads quality, annotating reads, annotating molecules, determining putative cells, and generating single‐cell expression matrix. The pipeline also determined the protein expression of every single cell via an algorithm according to the sequencing reads of Abseq library. Among all the output files, Matrix of UMI counts for each gene per cell was used for downstream analysis. Consortium Mouse Build 38(GRCm38) was used as a reference for BD pipeline.

For dimensionality reduction, clustering and visualization, Seurat was utilized for subsequent clustering analysis and visualization.^[^ ^37^ ^]^ Gene expression matrices for each sample were read and converted to Seurat objects. Cells with more than 25% mitochondrial UMI, less than 500 UMI or less than 200 genes were excluded from the downstream analysis. After a log‐normalization according to total cellular UMI count, principal components analysis (PCA) was performed based on the top 2 000 highly variable features after scaling the data with respect to UMI counts. Then, clustering was performed at resolution 0.6 and data was visualized using UMAP.

For cell type annotation, specific markers for each cluster were calculated using the FindAllMarkers function with the Wilcoxon Test under the following criteria: log2 fold change > 0.25; min. pct > 0.25. To unbiasedly identify the cell type in filtered sample datasets and the combined dataset, the R package SingleR (v1.4.1),^[^ ^38^ ^]^ a computational framework by reference to bulk transcriptomes helping to annotate cell type for each cluster, was used.

### 5.1 16S rRNA Gene Amplicon Sequencing and Analysis

Microbial DNA was extracted from mouse feces using the PF Mag‐Bind Stool DNA kit (Omega bio‐tek, Georgia, U.S.) according to the manufacturer's protocol. The quality and concentration of DNA were determined by 1% agarose gel electrophoresis and a NanoDrop^®^ ND‐2000 spectrophotometer (Thermo Scientific Inc., USA). For absolute quantification 16S rRNA amplicon sequencing, 12 different spike‐in sequences with four different concentrations (10^3^, 10^4^, 10^5^, and 10^6^ copies of internal standards) were added to the sample DNA pools. Spike‐in sequences consisted of conserved regions identical to those of selected natural 16S rRNA genes and artificial variable regions sharing negligible identity nucleotide sequences with the public databases, which worked like internal standard and facilitated the absolute quantification across samples. The hypervariable region V3‐V4 of the bacterial 16S rRNA genes were amplified with primer pairs 338F (5′‐ACTCCTACGGGAGGCAGCAG‐3′) and 806R (5′‐GGACTACHVGGGTWTCTAAT‐3′)^[^ ^39^ ^]^ by an ABI GeneAmp® 9700 PCR thermocycler (ABI, CA, USA). Amplification was conducted by combining 4 µL 5 × Fast Pfu buffer, 2 µL 2.5 mm dNTPs, 0.8 µL each primer (5 µm), 0.4 µL Fast Pfu polymerase, 0.2 µL BSA, 10 ng of template DNA, and ddH2O to a final volume of 20 µL. The conditions were as follows: 95 °C for 3 min, followed by 27 cycles of 95 °C for 30 s, 55 °C for 30 s, and 72 °C for 45 s, and 72 °C for 10 min. The amplicons were extracted from 2% agarose gel and purified using the AxyPrep DNA Gel Extraction Kit (Axygen Biosciences, Union City, CA, USA) according to the manufacturer's instructions and quantified using Quantus™ Fluorometer (Promega, USA).

Purified amplicons were pooled in equimolar amounts and paired‐end sequenced on an Illumina NovaSeq 6000 platform (Illumina, San Diego, USA) according to the standard protocols by Majorbio Bio‐Pharm Technology Co. Ltd. (Shanghai, China). Raw FASTQ files were de‐multiplexed using an in‐house perl script, and then quality‐filtered by fastp version 0.19.6^[^ ^40^ ^]^ and merged by FLASH version 1.2.7.^[^ ^41^ ^]^ The optimized sequences were clustered into operational taxonomic units (OTUs) with 97% sequence similarity level using UPARSE 7.1.^[^ ^42^ ^]^ The most abundant sequence for each OTU was selected as a representative sequence. The OTUs assigned to spike‐in sequences were filtered out and reads were counted. The standard curves (based on read counts vs spike‐in DNA copy number) for each sample were generated, and the quantitative abundance of each OTU in a sample was determined. The taxonomy of each OTU representative sequence was analyzed by RDP Classifier version 2.2^[^ ^43^ ^]^ against the 16S rRNA gene database (Silva v138) using a confidence threshold of 0.7, and then adjusted on the basis of the estimated rRNA operon copy number according to the *rrn*DB database.^[^ ^44^ ^]^ The spike‐in sequence was removed prior to subsequent analyses.

For 16S rRNA analysis, taxonomic classification at the class or family level was performed using R package vegan (version 2.4.3) software R language (version 3.3.1). Based on the OTUs information, rarefaction curves and alpha diversity indices Shannon index were calculated with Mothur v1.30.1.^[^ ^45^ ^]^ The similarity among the microbial communities in different samples was determined by principal coordinate analysis (PCoA) based on Bray‐curtis dissimilarity using Vegan v2.5‐3 package. The PERMANOVA test was used to assess the percentage of variation explained by the treatment along with its statistical significance using Vegan v2.5‐3 package. The correlations of Treg, ST2^+^ Treg, and ST2^−^ Treg frequency with gut microbiota at family level were determined by spearman correlation.

### Quantification of SCFAs

SCFAs were quantified by an ultra‐high performance liquid chromatography coupled with triple quadrupole mass spectrometry (1290 Infinity II UHPLC/6470A QQQ, Agilent) based on 3‐nitrophenylhydrazine (3‐NPH) derivatization as previous publications with modification.^[^ ^46^ ^]^ Frozen fecal samples of humans or mice were directly used for sample extraction and determination. The weighted frozen fecal samples of humans or mice were homogenized with tenfold volume (µL mg⁻^1^) of 50% aqueous acetonitrile containing internal standards (24 µg mL⁻^1^ of acetic‐d4 acid and propionic‐d6 acid, and 6 µg mL⁻^1^ butyric‐d7 acid) with zirconia beads at 40 Hz for 2 min (JX‐24, Jingxin, Shanghai, China). The homogenates were centrifuged (15000 g, 4 °C) for 15 min. Chemical derivatization was performed at 40 °C for 30 min after mixing supernatant (20 µL) with 50% aqueous acetonitrile (20 µL), 3‐NPH·HCl (200 mm, 20 µL), 1‐(3‐Dimethylaminopropyl)‐3‐ethylcarbodiimide hydrochloride (120 mm, 20 µL), and pyridine (6%, 20 µL). At last, the derivatization was stopped by an addition of 1% formic acid (20 µL) at room temperature for 5 min. The supernatant was applied to perform UHPLC‐MS/MS analysis with chromatographic separation by Waters Acquity UPLC BEH C18 column (2.1 mm × 100 mm, 1.7 µm) and optimized parameters of multiple reaction monitoring (MRM) mode. The concentrations of SCFAs in human or mouse fecal samples were calculated based on the standard curve of quantification for each analyte.

### Human Subjects

Patients who had been diagnosed with metabolic syndrome and subjected to bariatric surgery were recruited for the study. Stool samples were collected in tubes before the surgery and stored at −80 °C until metagenomic sequencing and quantification of SCFAs. Omental fat samples were collected in tubes with saline solution during surgery, placed in an ice box at 4 °C and transported directly to the laboratory. The stromal vascular fraction of omental fat was isolated and frozen at −80 °C before flow cytometry. For flow cytometry preparation, cells were resuscitated and then incubated in Roswell Park Memorial Institute (RPMI) 1640 medium supplemented with 10% fetal bovine serum, 1% GlutaMAX™ (Thermo Fisher Scientific), 1% HEPES (Thermo Fisher Scientific) and 1% streptomycin–penicillin at 37 °C under 5% CO2 for 40 min. Single‐cell suspensions were prepared after centrifugation. The exclusion criteria include: (1) patients with previous diseases (such as cancer, autoimmune diseases, etc). (2) For quantification of SCFAs, the stool samples were insufficient for detection. (3) For flow cytometry of omental fat, cells with poor activity and less than 500 lymphocytes were excluded. All participants provided written informed consent as approved by the ethics commission of Shanghai Ninth People's Hospital, Shanghai Jiao Tong University School of Medicine (SH9H‐2022‐T343‐2). All patient studies were carried out in accordance with the Declaration of Helsinki.

### Metagenomic Sequencing and Analysis

Fecal DNA from mouse samples was extracted using the QIAamp PowerFecal Pro DNA Kit (QIAGEN) and then fragmented an average size of ≈400 bp using Covaris M220 (Gene Company Limited, China). Paired‐end library was constructed using NEXTFLEX Rapid DNA‐Seq (Bioo Scientific, Austin, TX, USA) and sequenced on an Illumina NovaSeq™ X Plus platform (Illumina Inc., San Diego, CA, USA). Raw reads were trimmed of adapters, and low‐quality reads were removed by fastp (version 0.20.0).^[^ ^47^ ^]^ Reads were aligned to the Mus musculus genome by BWA (version 0.7.17),^[^ ^48^ ^]^ and any hits associated with the reads and their mated reads were removed. The quality‐filtered data were assembled using MEGAHIT (version 1.1.2),^[^ ^49^ ^]^ and open reading frames (ORFs) from each assembled contigs were predicted using Prodigal (version 2.6.3)^[^ ^50^ ^]^ and a length ≥100 bp ORFs were retrieved. A non‐redundant gene catalog was constructed using CD‐HIT (version 4.7),^[^ ^51^ ^]^ and gene abundance was estimated by SOAPaligner (version soap2.21release)^[^ ^52^ ^]^ with 95% identity. Taxonomic classification of non‐redundant genes was obtained by aligning them against the NCBI NR database (version August 2023) by DIAMOND (version 2.0.13)^[^ ^53^ ^]^ using an e‐value cutoff of 1e‐5. Differential analysis at the species level was performed using the Kruskal‐Wallis test. Spearman correlation analysis was used to assess the correlations between Treg frequency and gut microbiota at species level.

For human samples, fecal DNA from humans was extracted using MagPure Stool DNA KF Kit B (MAGEN, Guangzhou, China)^[^ ^54^ ^]^ following the manufacturer's protocol. Library preparation was performed using MGIEasy Universal DNA Library Prep Set (MGI‐Shenzhen, China). The resulting libraries were sequenced on the DNBSEQ‐T7 platform (BGI‐Shenzhen, China), generating sequencing reads of PE100 bases length. An adapter was trimmed and low‐quality reads were filtered using fastp (version 0.23.4).^[^ ^47^ ^]^ Then, host sequences were removed by aligning sequencing reads back to the host genome reference (hg38) using Bowtie2 (version 2.5.1).^[^ ^55^ ^]^ Taxonomic profiling of the metagenomic samples was performed using MetaPhlAn (version 4.0.6)^[^ ^56^ ^]^ which used clade‐specific markers to provide pan‐microbial (bacterial, archaeal, viral, and eukaryotic) quantification at species‐level. MetaPhlAn was run with parameters “–read_min_len 50 –force –no_map –add_viruses”. The relationships between microbial data and clinical indicators were analyzed using the Spearman correlation with the stats package. Heatmaps were generated to visualize the correlations with the ComplexHeatmap^[^ ^57^ ^]^ package in R version 4.4.0.

### Statistical Analysis

Statistical analysis was performed with GraphPad Prism 9.0. Unless otherwise noted, comparisons between two groups were determined by two‐tailed unpaired Student's *t*‐tests or Mann‐Whitney test. Unless otherwise noted, comparisons between multiple groups, *P* values were calculated by Kruskal‐Wallis test with Dunn's multiple comparisons test, one‐way ANOVA with Dunnett's multiple comparisons test or Šídák's multiple comparisons test, and two‐way ANOVA with Šidák's multiple‐comparisons test, as indicated in each figure legend. Two‐way repeated‐measures ANOVA was used for comparisons of two curves with multiple time points. All experiments were repeated at least two or three times. Error bars show the mean ± s.e.m. *P* values are represented in figures as follows: *p < 0.05, **p <  0.01, ***p < 0.001, ****p < 0.0001.

## Conflict of Interest

The authors declare no conflict of interest.

## Author Contributions

R.L. and W.W. conceived and designed the project. B. C. and L. G. performed most of the experiments and data analysis. L.W. helped to isolate the stromal vascular fraction of human VATs. C.W., Y.G., Z.C., and M.Z. performed some of the animal experiments. Y.L. performed SPR experiments. Y.C., C.Y., and B.W. contributed to the human adipose tissue collection. B.L., Y.B., G.N., and J.W. contributed to the data discussion. B.C., L.G., and R.L. wrote the manuscript with contributions from all the authors.

## Supporting information



Supporting Information

## Data Availability

All data generated and supporting the findings of this study are available within the paper. The single‐cell RNA sequencing data have been deposited in the Gene Expression Omnibus (GEO) under accession number GSE240739. The 16S rRNA amplicon sequencing data and metagenomic sequencing data from mouse fecal samples have been submitted to the Sequence Read Archive (SRA), with the accession numbers PRJNA1004751 and PRJNA1224995, respectively. The human fecal metagenomic sequencing data have been deposited in the Genome Sequence Archive of the Beijing Institute of Genomics, Chinese Academy of Sciences (https://bigd.big.ac.cn/gsub/) under the accession number PRJCA036095. Additional information and materials will be made available upon request.
