# Supplementary material for: Gut Microbiota‐Butyrate‐PPARγ Axis Modulates Adipose Regulatory T Cell Population
Source: Adv Sci (Weinh). 2025 Feb 25;12(20):2411086. doi: 10.1002/advs.202411086 (PMC12120792; doi:10.1002/advs.202411086)
Supplement: Supplementary file 1 — Supporting Information [file ADVS-12-2411086-s001.docx]

Supporting Information

**Gut Microbiota-Butyrate-PPARγ Axis Modulates Adipose Regulatory T Cell Population**

*Banru Chen, Lizhi Guan, Chao Wu, Yiwen Gong, Lei Wu, Minchun Zhang, Zhiwen Cao, Yufei Chen, Chengcan Yang, Bing Wang, Yunqi Li, Bin Li, Yufang Bi, Guang Ning, Jiqiu Wang, Weiqing Wang*, Ruixin Liu**


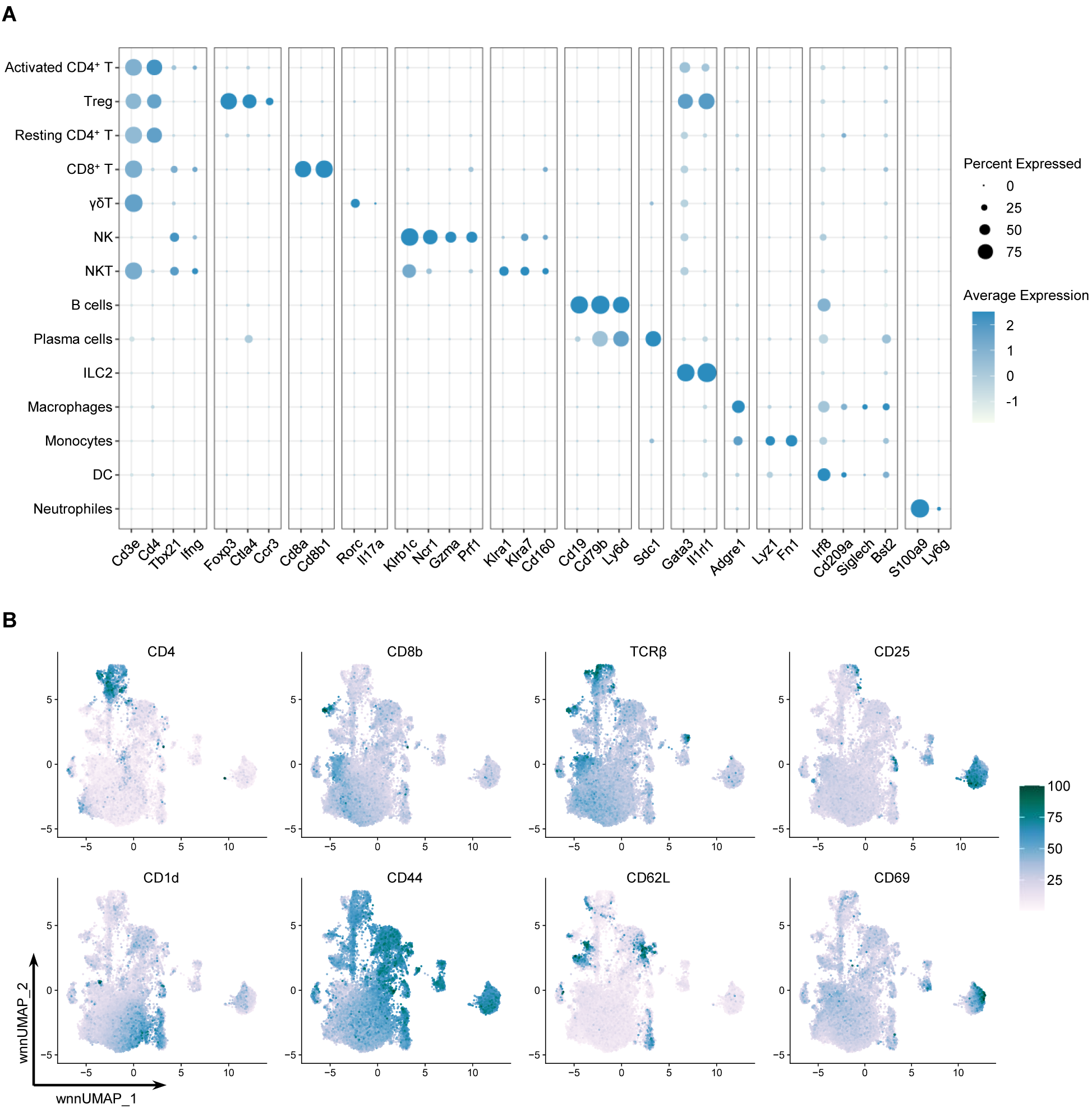


**Figure S1.** Single-cell transcriptomes of VAT immune cells. A) Dot plots showing the expression of specific marker genes in different cell types. B) UMAP plots indicating expression of CD4, CD8b, TCRβ, CD25, CD1d, CD44, CD62L, and CD69 in VAT CD45^+^ cells.


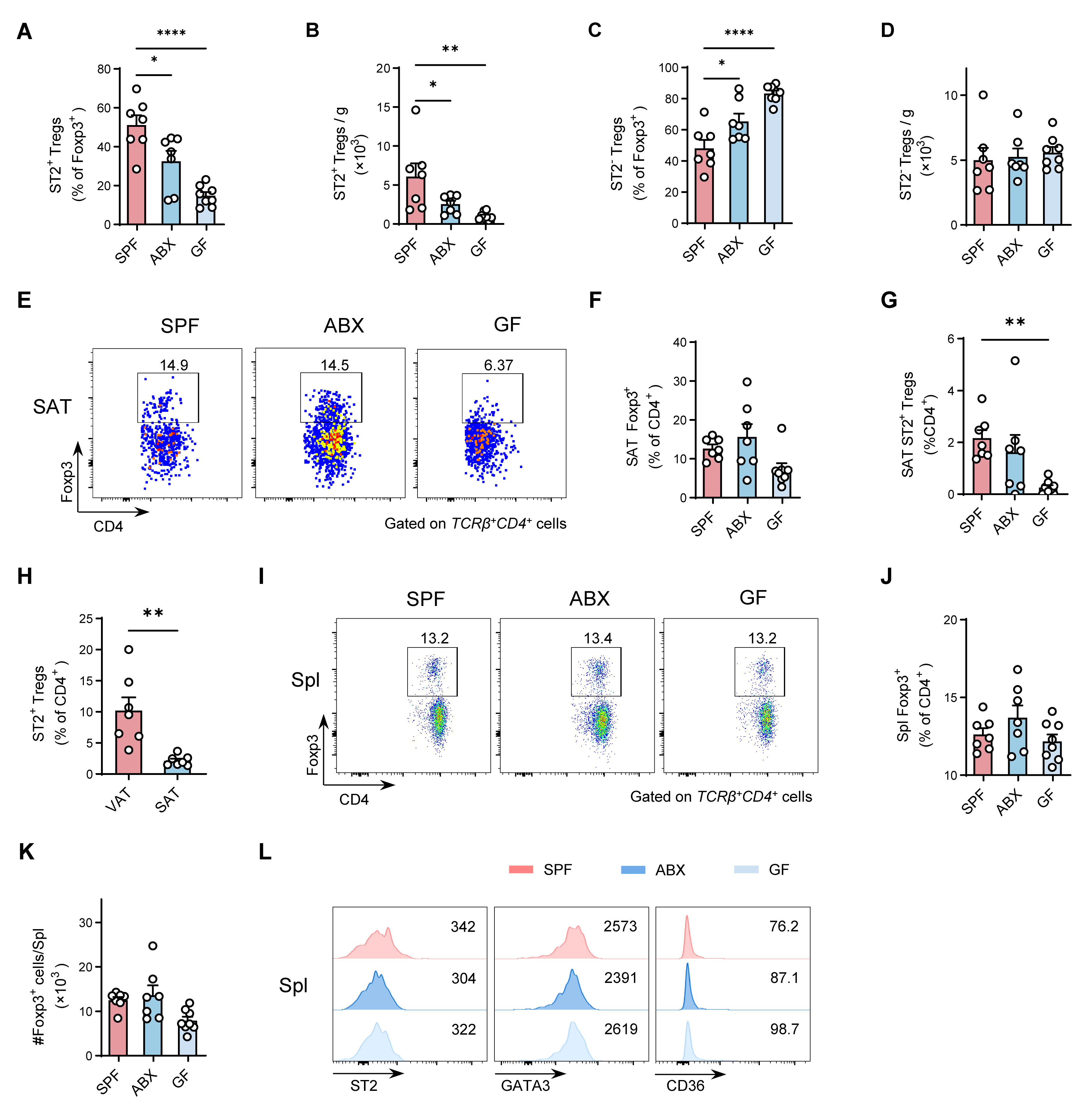


**Figure S2.** Flow cytometry analysis for VAT Treg cells, SAT Treg cells and splenic Treg cells after gut microbiota deletion. A) The percentages of ST2^+^ Treg cells among VAT Foxp3^+^ Treg cells from 15-week-old SPF (*n*=7), ABX (*n*=7), and GF (*n*=8) mice. B) Number of ST2^+^ Treg cells per gram VAT of the three groups. C) The percentages of ST2^-^ Treg cells among VAT Foxp3^+^ Treg cells of the three groups. D) Number of ST2^-^ Treg cells per gram of VAT in the three groups. E) Representative flow-cytometric plots of Foxp3^+^ Treg cells among TCRβ^+^CD4^+^ cells in subcutaneous adipose tissue (SAT) of indicated groups. Numbers in the plots indicate the percentages of Foxp3^+^ Treg cells among SAT TCRβ^+^CD4^+^cells. F) The percentages of Foxp3^+^ Treg cells among CD4^+^ cells in SAT of SPF, ABX and GF mice. G) The percentages of ST2^+^ Treg cells among CD4^+^ cells in SAT of the three groups. H) The percentages of ST2^+^ Treg cells among CD4^+^ cells in SAT and VAT from SPF mice. Data are derived from Figure 1 J - SPF group and Figure S2G - SPF group. I) Representative flow-cytometric plots of Foxp3^+^ Treg cells among TCRβ^+^ CD4^+^ cells in spleen. Numbers in the plots indicate the percentages of Foxp3^+^ Treg cells among TCRβ^+^ CD4^+^ cells in the spleen of SPF, ABX, and GF mice. J and K) The percentages of Foxp3^+^ Treg cells among CD4^+^ cells (J) and number of Foxp3^+^ Treg cells (K) in spleen of the three groups. L) Representative flow-cytometric histograms of indicated cell markers ST2, GATA3, CD36 in spleen Foxp3^+^ Treg cells. Numbers in the histograms indicate the MFI of these indicated markers among Foxp3^+^ Treg cells. Data are shown as the mean ± s.e.m. Statistical differences between groups were assessed by one-way ANOVA with Dunnett’s multiple comparisons test (A-D, F, G, J, K) and two-tailed unpaired Student's t-tests (H). **p* < 0.05, ***p* < 0.01, ****p* < 0.001, *****p* < 0.0001.


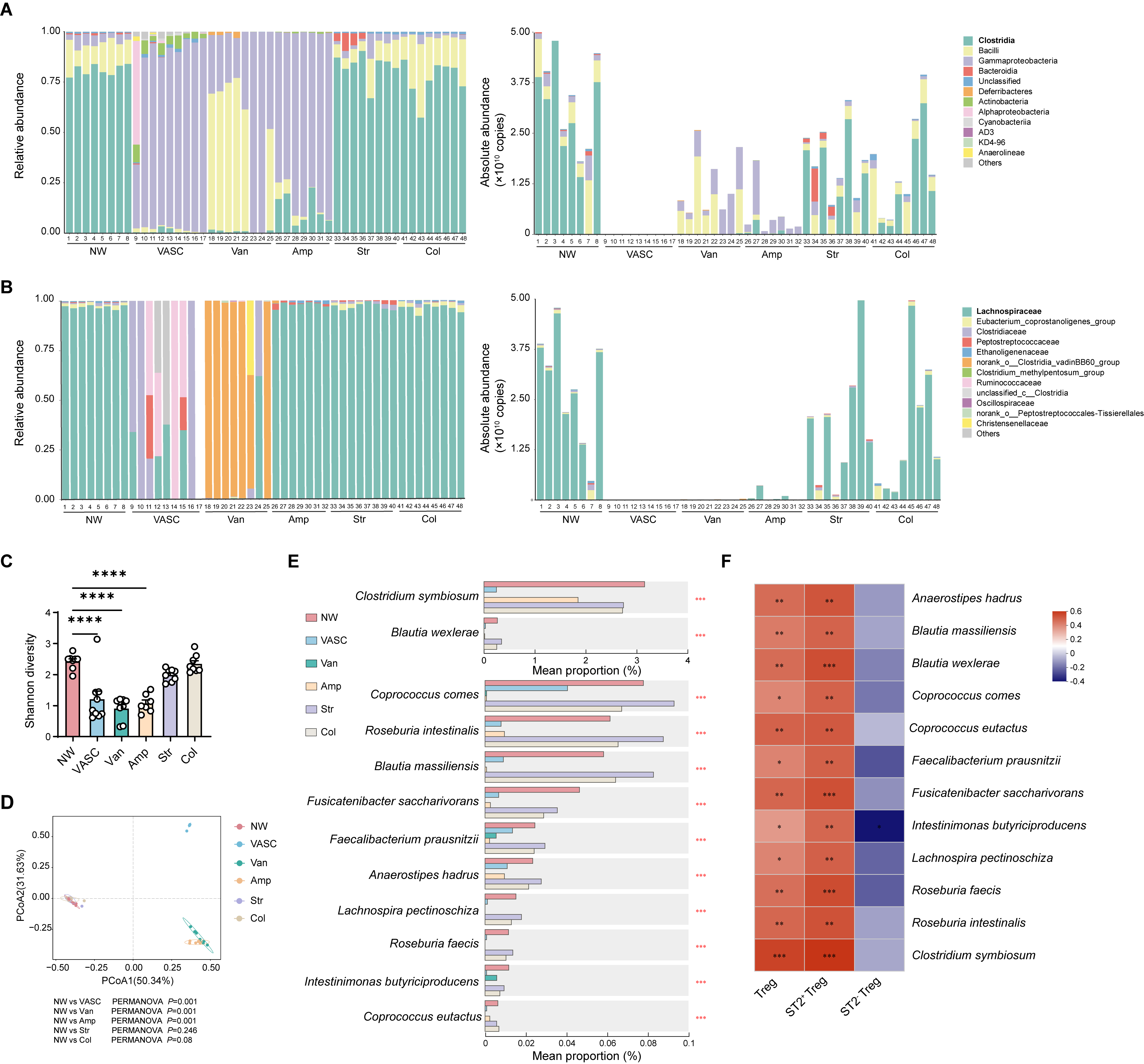


**Figure S3.** The alterations of gut microbiota composition after different antibiotic treatment. **A, B**) Relative abundance (left) and absolute abundance (right) of bacterial taxa at the class level (**A**) or the family level (**B**) in each individual mouse through 16S rRNA analysis. **C**) Alpha diversity in each group as determined by Shannon diversity index. **D**) Beta diversity of each group as determined by principal coordinates analysis (PCoA) of Bray-Curtis dissimilarity (PERMANOVA: NW vs VASC *R^2^*=0.719, *P*=0.001; NW vs Van *R^2^*=0.628, *P*=0.001; NW vs Amp *R^2^*=0.629, *P*=0.001; NW vs Str *R^2^*=0.0866, *P*=0.246; NW vs Col *R^2^*=0.150, *P*=0.08). **E**) Differential abundance of species that are reported to produce butyrate among different groups, as determined by metagenomic sequencing. **F**) Heatmap showing Spearman’s correlation coefficients between butyrate-producing species and the percentages of Treg, ST2^+^ Tregs and ST2^-^ Tregs among CD4^+^ cells. Data are shown as the mean ± s.e.m. (C) Statistical differences between groups were assessed by one-way ANOVA with Dunnett’s multiple comparisons test (C) and the Kruskal-Wallis test (**E**). **p* < 0.05, ***p* < 0.01, ****p* < 0.001, *****p* < 0.0001.


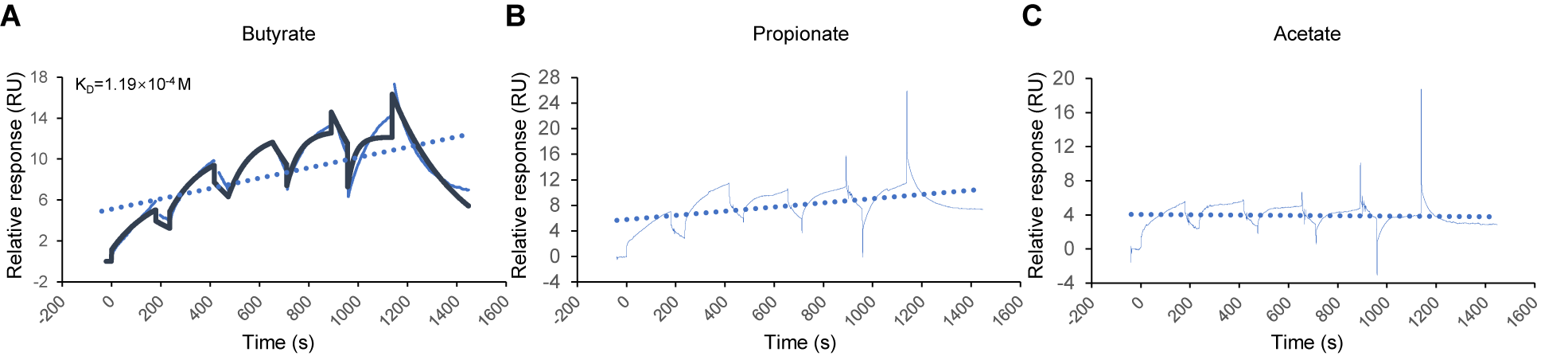


**Figure S4.** Butyrate, but not propionate and acetate, binds to PPARγ. Sensorgrams for butyrate (A), propionate (B), acetate (C) with PPARγ through surface plasmon resonance (SPR) assays.

**
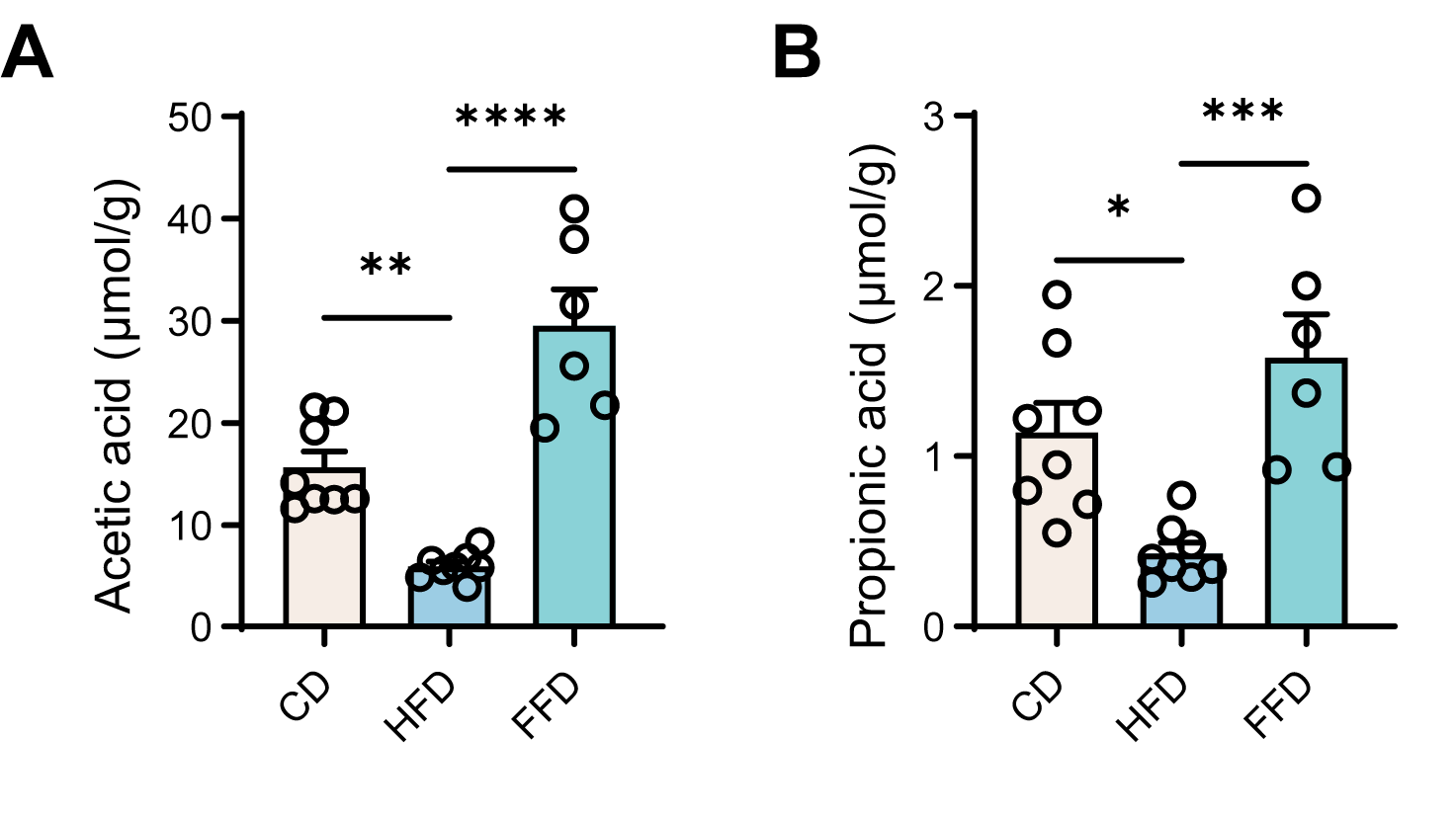
**

**Figure S5.** Acetic acid and propionic acid concentrations in fecal samples of mice treated with each diet. Data are shown as the mean ± s.e.m. Statistical differences between groups were assessed by one-way ANOVA with Šídák's multiple comparisons test. **p* < 0.05, ***p* < 0.01, ****p* < 0.001, *****p* < 0.0001.


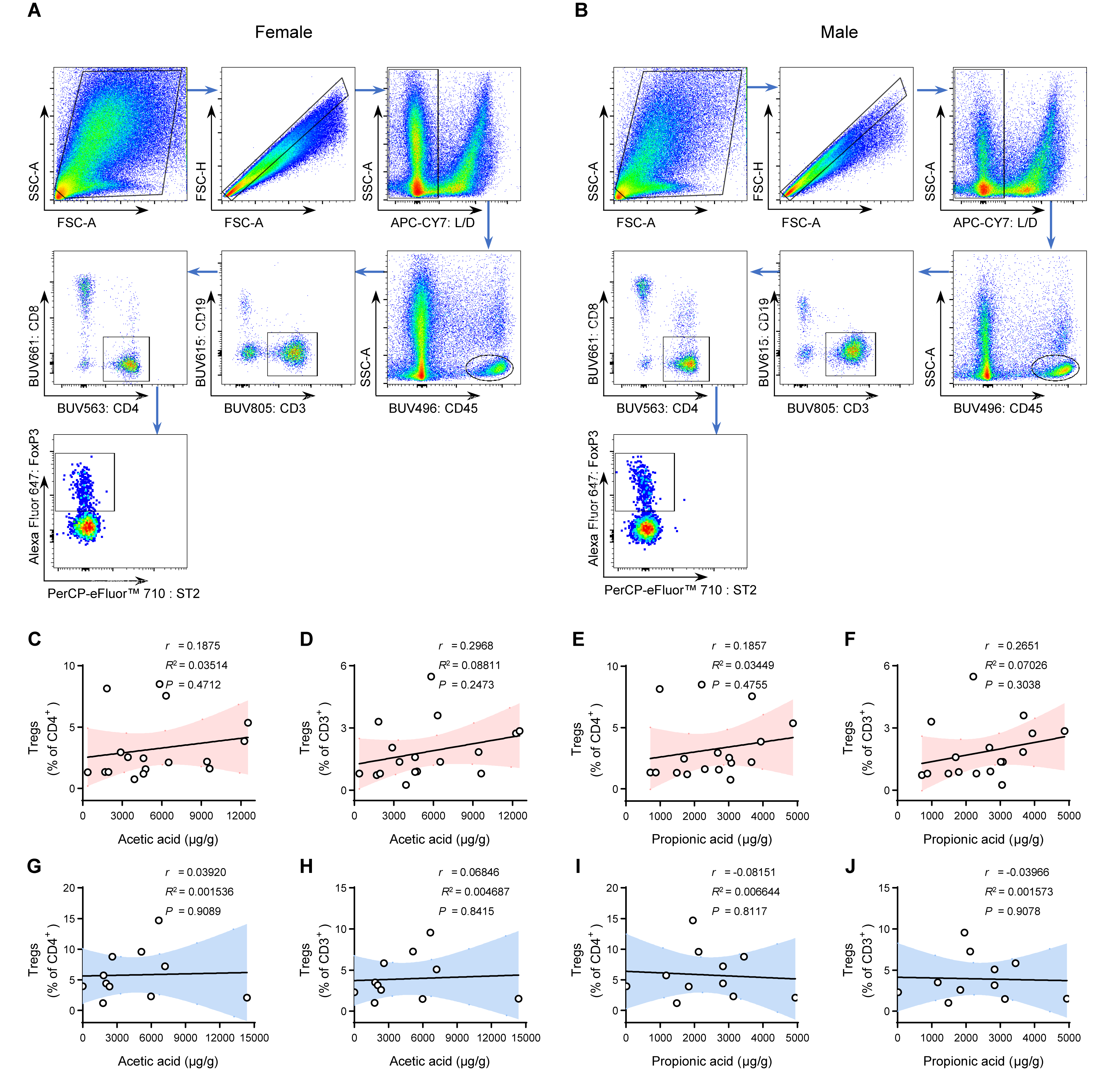


**Figure S6.** Gating strategy for Treg cells and their correlations to fecal SCFAs in human omental fat. A, B) Gating strategy for detecting Treg cells (CD4^+^ Foxp3^+^) in human VAT from female (A) and male (B) obese subjects. C, D) Correlations between fecal concentration of acetic acid and the percentages of Treg cells among CD4^+^ cells (C) and among CD3^+^ cells (D) in female omental fat. E, F) Correlations between fecal concentration of propionic acid and the percentages of Treg cells among CD4^+^ cells (E) and among CD3^+^ cells (F) in female omental fat. G, H) Correlations between fecal concentration of acetic acid and the percentages of Treg cells among CD4^+^ cells (G) and among CD3^+^ cells (H) in male omental fat. I, J) Correlations between fecal concentration of propionic acid and the percentages of Treg cells among CD4^+^ cells (I) and among CD3^+^ cells (J) in male omental fat. *n*=17 for female obese subjects; *n*=11 for male obese subjects. The colored area indicates 95% confidence intervals. *P* values were determined by simple linear regression and Pearson’s correlation.


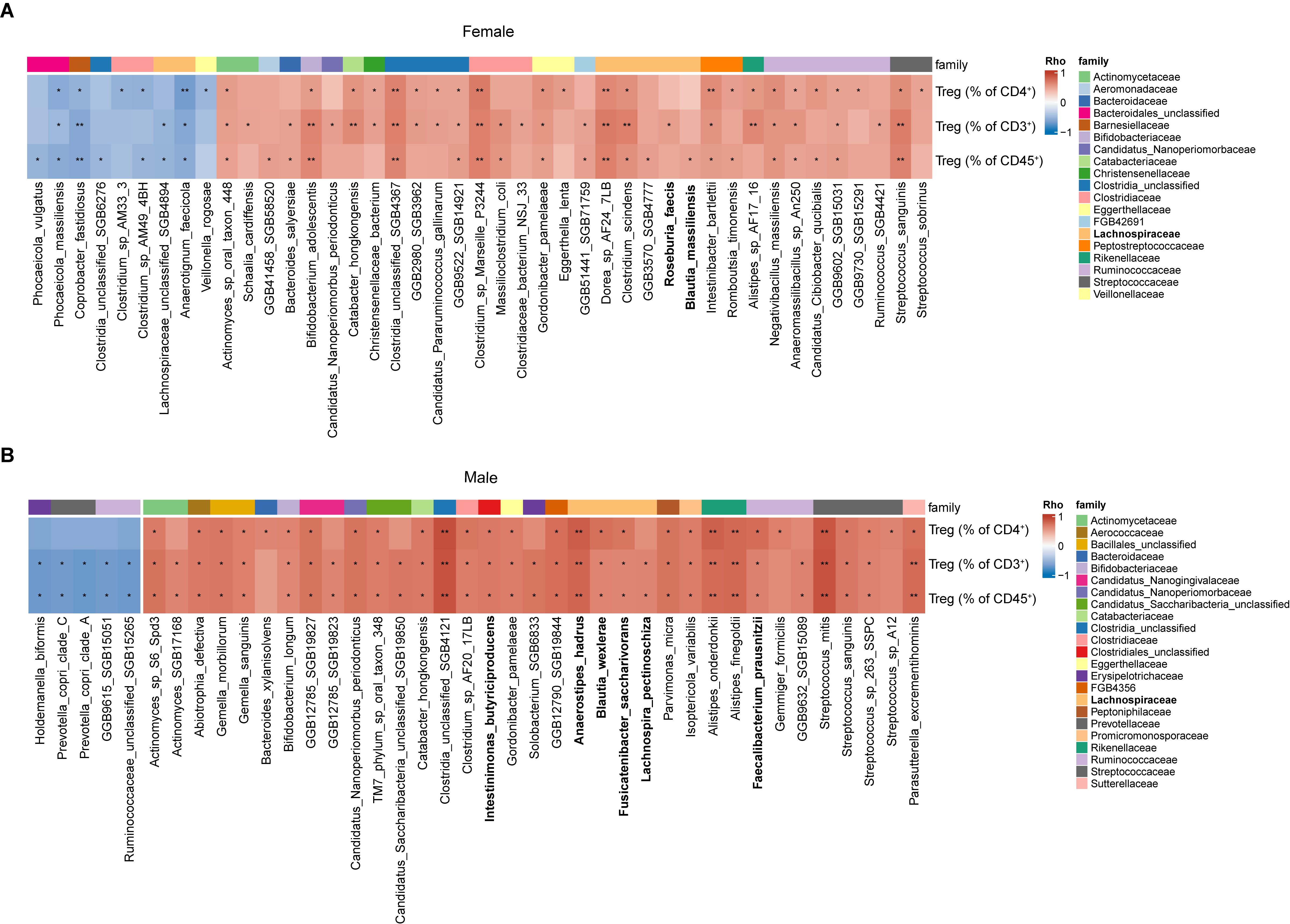


**Figure S7.** The correlations between gut microbial species and human VAT Treg cells. A and B) Heatmap illustrating the associations between fecal microbial species and the percentages of Treg among CD4^+^ cells, CD3^+^ cells or CD45^+^ cells in female (A) and male (B) participants. Only species correlated with at least one Treg percentage and with *P* < 0.05 are shown. Species that can produce butyrate are shown in boldface. Female, n=17; Male, n=10. Correlations were assessed using Spearman’s rank correlation. Statistical significance is indicated as **p* < 0.05, and ***p*< 0.01.

**Table S1.** The composition of CD, HFD and FFD used in this study.

| Product # | CD, D12450J | | HFD, D12492 | | FFD, RD21030802 | |
| --- | --- | --- | --- | --- | --- | --- |
|  | 10 kcal% Fat | | 60 kcal% Fat | | Diet with 60 kcal% Fat and fiber from Inulin | |
|  |  |  |  |  |  |  |
|  | gm% | kcal% | gm% | kcal% | gm% | kcal% |
| Protein | 16.9 | 20.0 | 26.2 | 20.0 | 22.9 | 20.0 |
| Carbohydrate | 67.3 | 70.0 | 26.3 | 20.1 | 18.8 | 20.1 |
| Fat | 4.3 | 10.0 | 34.9 | 59.9 | 30.4 | 59.9 |
| Total |  | 100.0 |  | 100.0 |  | 100.0 |
| kcal/gm | 3.85 |  | 5.24 |  | 4.58 |  |
|  |  |  |  |  |  |  |
| Ingredient | gm | kcal | gm | kcal | gm | kcal |
| Casein | 200 | 800 | 200 | 800 | 200 | 800 |
| L-Cystine | 3 | 12 | 3 | 12 | 3 | 12 |
|  |  |  |  |  |  |  |
| Corn Starch | 506.2 | 2024.8 | 0 | 0 | 0 | 0 |
| Maltodextrin 10 | 125 | 500 | 125 | 500 | 88 | 352 |
| Sucrose | 68.8 | 275.2 | 68.8 | 275.2 | 68.8 | 275.2 |
|  |  |  |  |  |  |  |
| Cellulose, BW200 | 50 | 0 | 50 | 0 | 50 | 0 |
|  |  |  |  |  |  |  |
| inulin |  |  |  |  | 150 | 150 |
|  |  |  |  |  |  |  |
| Soybean Oil | 25 | 225 | 25 | 225 | 25 | 225 |
| Lard | 20 | 180 | 245 | 2205 | 245 | 2205 |
|  |  |  |  |  |  |  |
| Mineral Mix S10026 | 10 | 0 | 10 | 0 | 10 | 0 |
| DiCalcium Phosphate | 13 | 0 | 13 | 0 | 13 | 0 |
| Calcium Carbonate | 5.5 | 0 | 5.5 | 0 | 5.5 | 0 |
| Potassium Citrate, 1 H2O | 16.5 | 0 | 16.5 | 0 | 16.5 | 0 |
|  |  |  |  |  |  |  |
| Vitamin Mix V10001 | 10 | 40 | 10 | 40 | 10 | 40 |
| Vitamin Mix V10001C |  |  |  |  |  |  |
| Choline Bitartrate | 2 | 0 | 2 | 0 | 2 | 0 |
| FD&C Yellow Dye #5 | 0.04 | 0 | 0 | 0 | 0 | 0 |
| FD&C Red Dye #40 | 0 | 0 | 0 | 0 | 0.05 | 0 |
| FD&C Blue Dye #1 | 0.01 | 0 | 0.05 | 0 | 0 | 0 |
|  |  |  |  |  |  |  |
| Total | 1055.05 | 4057 | 773.85 | 4057 | 886.85 | 4059 |

**Table S2.** Primer sequences used in this study.

| Gene |  | Sequence |
| --- | --- | --- |
| *Gapdh* | Forward | ACGGCCGCATCTTCTTGTGCA |
|  | Reverse | AATGGCAGCCCTGGTGACCA |
| *Pparg* | Forward | TCACAAGAGCTGACCCAATG |
|  | Reverse | TGAGGCCTGTTGTAGAGCTG |
| *Rantes* | Forward | GGAATTCGGGTACCATGAAGATCTC |
|  | Reverse | GCGGATCCTAGCTCATCTCCAAATA |
| *Mcp1* | Forward | GGAATTCACCACCATGCAGGTCCCTGTC |
|  | Reverse | GCGGATCCGAGTCACACTAGTTCACT |
| *Mip1a* | Forward | GGAATTCACCATGAAGGTCTCCACCACTG |
|  | Reverse | GCGGATCCAAGACTCTCAGGCATTC |
| *Il1b* | Forward | CTGCAGCTGGAGAGTGTGGAT |
|  | Reverse | GGGAACTCTGCAGACTCAAACT |
| *Tnfa* | Forward | CCCTCACACTCAGATCATCTTCT |
|  | Reverse | GCTACGACGTGGGCTACAG |
| *Universal* | Forward | ACTCCTACGGGAGGCAGCAGT |
|  | Reverse | ATTACCGCGGCTGCTGGC |
| *butyryl-CoA: acetate CoA-transferase* | Forward | GCIGAICATTTCACITGGAAYWSITGGCAYATG |
|  | Reverse | CCTGCCTTTGCAATRTCIACRAANGC |
